# Pharmacogenomic and clinical data link non-pharmacokinetic metabolic dysregulation to drug side effect pathogenesis

**DOI:** 10.1038/ncomms8101

**Published:** 2015-06-09

**Authors:** Daniel C. Zielinski, Fabian V. Filipp, Aarash Bordbar, Kasper Jensen, Jeffrey W. Smith, Markus J. Herrgard, Monica L. Mo, Bernhard O. Palsson

**Affiliations:** 1 Department of Bioengineering, University of California, San Diego, La Jolla, California 92093-0412, USA; 2 Cancer Research Center, Sanford-Burnham Institute for Medical Research, 10901 North Torrey Pines Road, La Jolla, California 92037, USA; 3 UC Merced, Quantitative and Systems Biology, University of California Merced, 5200 North Lake Road, Merced, California 95343, USA; 4 Sinopia Biosciences, 600 W Broadway Suite 700, San Diego, CA 92101, USA; 5 Center for Biological Sequence Analysis, Department of Systems Biology, Technical University of Denmark, Kemitorvet, Building 208, Lyngby DK-2800, Denmark; 6 Novo Nordisk Foundation Center for Biosustainability, Technical University of Denmark, Kogle Alle 6, Horshølm 2970, Denmark; 7 Department of Pediatrics, University of California, San Diego, La Jolla, California 92093-0412, USA

## Abstract

Drug side effects cause a significant clinical and economic burden. However, mechanisms of drug action underlying side effect pathogenesis remain largely unknown. Here, we integrate pharmacogenomic and clinical data with a human metabolic network and find that non-pharmacokinetic metabolic pathways dysregulated by drugs are linked to the development of side effects. We show such dysregulated metabolic pathways contain genes with sequence variants affecting side effect incidence, play established roles in pathophysiology, have significantly altered activity in corresponding diseases, are susceptible to metabolic inhibitors and are effective targets for therapeutic nutrient supplementation. Our results indicate that metabolic dysregulation represents a common mechanism underlying side effect pathogenesis that is distinct from the role of metabolism in drug clearance. We suggest that elucidating the relationships between the cellular response to drugs, genetic variation of patients and cell metabolism may help managing side effects by personalizing drug prescriptions and nutritional intervention strategies.

## Abstract (web summary)

Adverse drug reactions are an important clinical problem. Here the authors combine information about drug-induced gene expression changes and genetic variability of patients with a genome-scale metabolic model to identify drug-induced changes in cellular metabolism that may be linked to drug side effects.

## Graphical abstract

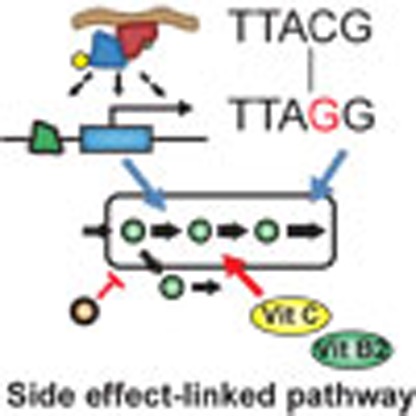

Adverse drug reactions, commonly known as side effects, are thought to be responsible for as much as 11% of hospital admissions^1^,^2^, a fifth of both phase II^3^ and III^4^ clinical trial failures, high-profile market withdrawals (for example, Vioxx, Lipobay), and a large fraction of patient therapeutic non-compliance incidents^5^. Risk factors associated with side effects have been identified, including number of drugs prescribed^6^, patient age^7^ and genetic variants^8^. Side effect-linked genetic variants identified so far are predominantly associated with drug pharmacokinetics, thereby affecting exposure of the body to a particular drug, but these variants do not give any indication of the mechanism by which pathogenesis is initiated. A recent study suggests that as many as half of drug side effects are related to known drug–protein-binding events^9^, and progress has been made towards systematically identifying drug-binding events^10^. However, only modest progress has been made towards elucidating specific drug-induced changes downstream of binding events for the majority of drugs (Fig. 1a)^11^. These downstream effects in many cases may be most directly tied to side effect pathogenesis as well as patient genetic and environmental background.

Recent literature suggests that altered gene expression induced by drug treatment may be one mechanism by which drugs induce systemic off-target effects^12^,^13^,^14^,^15^. Unfortunately, the lack of clinical data has impeded the determination of causality of particular gene expression changes in side effect pathogenesis^16^. Recent studies have successfully utilized *in vitro* drug-treated gene expression profiles to predict clinical drug effectiveness^17^,^18^, suggesting that *in vitro* data may contain features that are clinically conserved. However, demonstrating the relevance of *in vitro* drug response features to clinical side effect pathogenesis presents a significant challenge, due largely to the lack of suitable validating data sets and difficulty of clinical experimentation.

To address this challenge, we develop a network-based data analysis workflow built upon the use of *in vitro* drug treatment data to identify candidate side effect-linked features and a large collection of historical clinical and disease model data as a source of validation (Fig. 1). First, we identify *in vitro* gene expression changes preferentially induced by drugs with clinically defined side effects to identify candidate side effect-linked expression features. Then, we cross-reference these side effect-linked features with independent legacy clinical data found in the literature to corroborate their relevance in terms of five causal relationships. We implement this strategy within the context of the reconstructed global human metabolic network^19^,^20^, which provides a biologically coherent structure for data integration due to the high degree of network annotation and clear functional connectivity between genes via metabolic pathways^20^,^21^.

## Results

### Calculation of drug-induced metabolite perturbations

**Figure 1:**
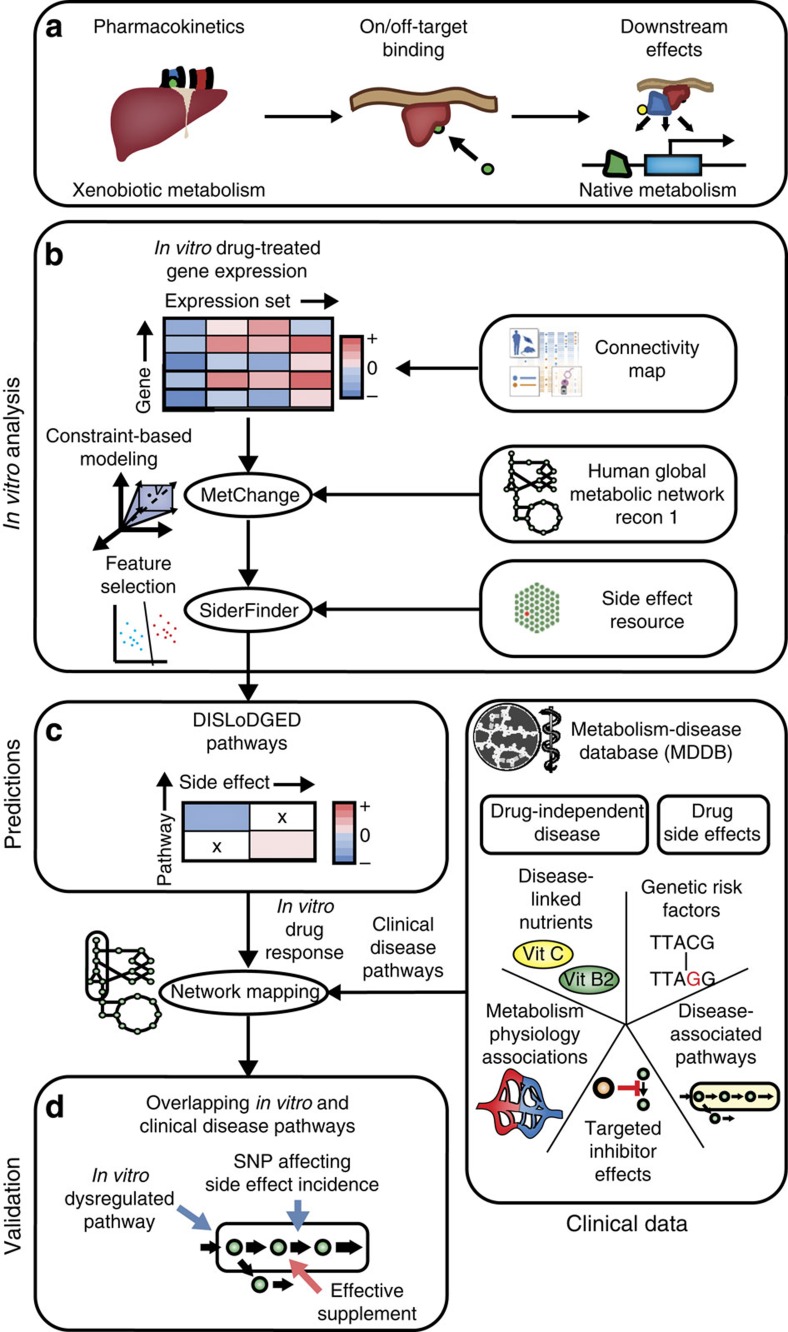
Overview and workflow used in this study. (**a**) Studies examining side effect pathogenesis focus primarily on drug pharmacokinetics, involving drug transport and clearance, and drug binding in terms of on and off target-binding events. This study examines potential pathogenic mechanisms related to transcriptional changes downstream of clearance and binding events. (**b**) Drug-treated gene expression profiles from the Connectivity Map database are analysed in the context of the metabolic network reconstruction Recon 1 using constraint-based modelling to identify drug-induced pathway expression changes. Drug-induced metabolic pathway expression changes are analysed in terms of drug side effects from the Side Effect Resource (SIDER) using a feature selection genetic algorithm to determine metabolic pathway perturbations conserved in particular side effects, termed DISLoDGED pathways. (**c**) A new database, the Metabolism Disease Database (MDDB), was generated by manual curation of literature to establish links between altered metabolic pathway function and pathologies, and this database was used to analyse DISLoDGED metabolic pathways. (**d**) Five candidate causal mechanisms for metabolic changes in side effect pathogenesis (listed in the MDDB panel) are assessed in a large-scale manner by comparing these *in vitro* perturbations to clinical data linking particular metabolic pathways to disease.

**Figure 2:**
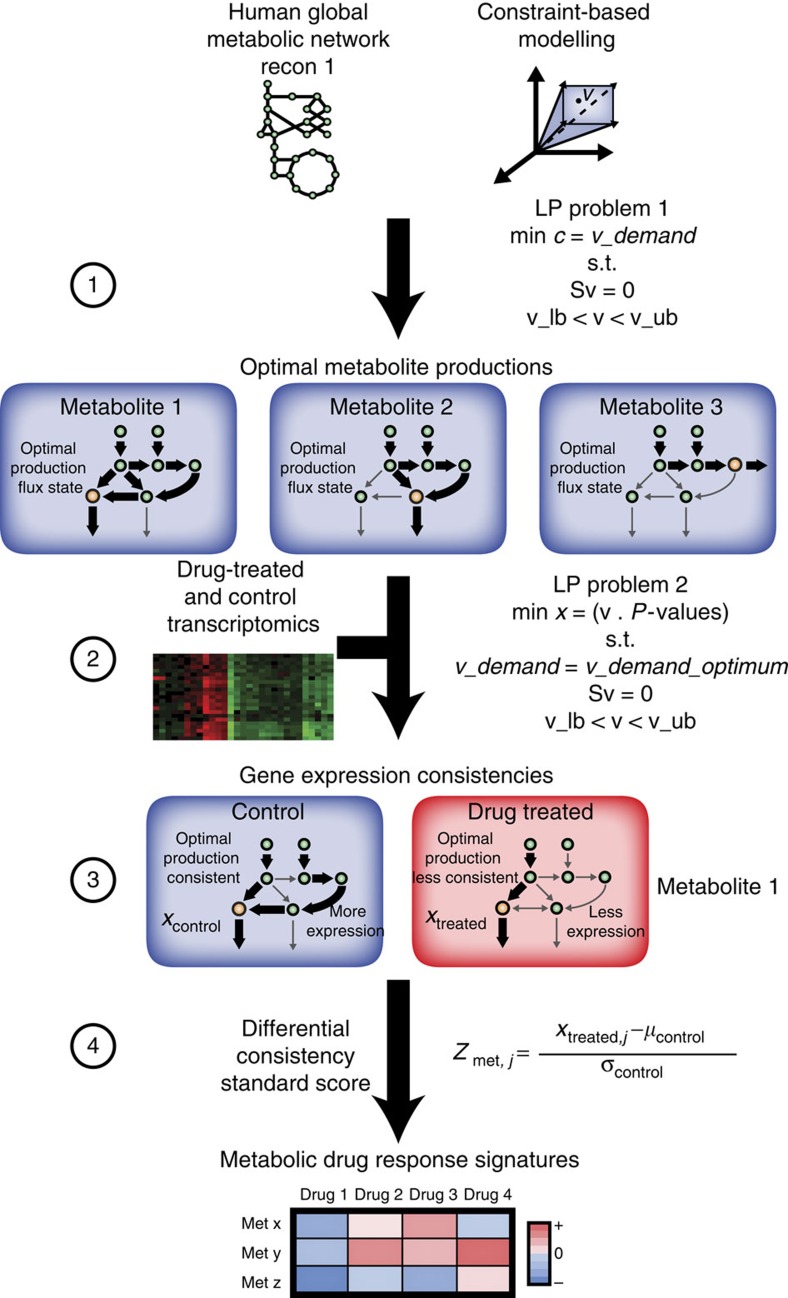
Description of the MetChange algorithm. (1) Using a metabolic network reconstruction, sink (demand) reactions are added for each metabolite. Demand reactions are irreversible with the stoichiometry: metabolite −> Ø. Each demand reaction is maximized in turn to obtain maximal production values for each metabolite using a linear programming problem (LP Problem 1). (2) Reaction presence/absence *P*-values are generated from gene expression data and mapped onto the metabolic network. A second linear programming problem is then solved (LP Problem 2) for each metabolite. LP Problem 2 identifies the flux solution that minimizes the inconsistency of the gene expression data with the optimal production of a metabolite by restricting the demand reaction for the metabolite to be at maximal flux, and subsequently minimizing an inconsistency score of (*v* × *P*-values). (3) An example case for metabolite 1. It is observed that the control data have greater expression (lower presence/absence *P*-value) for certain production reactions. Greater expression of production reactions results in a lower production inconsistency score for the control gene expression sample, compared with the drug-treated case, in which certain production reactions are less expressed (higher presence/absence *P*-value). (4) As different metabolites have different combinations of production reactions, they cannot be compared directly within samples. Instead, scores are compared for the same metabolite between control and treated samples to generate differential consistency scores using a simple standard score. Once standardized, metabolites can be compared within drugs to identify regions where perturbation in production potential has occurred due to gene expression changes.

We first identified drug-induced metabolic gene expression changes within 6,040 gene expression profiles in the Connectivity Map (CMap) data set, representing three human cell lines exposed to 1,221 drug compounds^22^ (Fig. 1a). We analysed the expression profiles using the reconstructed global human metabolic network Recon 1 (ref. 19) with a novel metabolic pathway analysis algorithm, termed MetChange (Metabolite-Centered Hotspots of Altered Network Gene Expression). MetChange is a constraint-based modelling^23^ algorithm that computes a score for each metabolite summarizing the drug-induced gene expression changes along calculated production pathways for the metabolite (Fig. 2). A MetChange score for a metabolite defines how expression has changed in a pathway containing these metabolite production reactions. Production in this case does not necessarily indicate secretion, as the majority of metabolites produced by one metabolic pathway are consumed in other metabolic pathways. We also note that gene expression is not the sole determinant of pathway activity, as gene and protein expression are imperfectly correlated and enzyme functional state may change due to perturbation as well. However, change in metabolic gene expression may still indicate a pathogenic metabolic functional change.

### Validation of calculated metabolic perturbations

**Figure 3:**
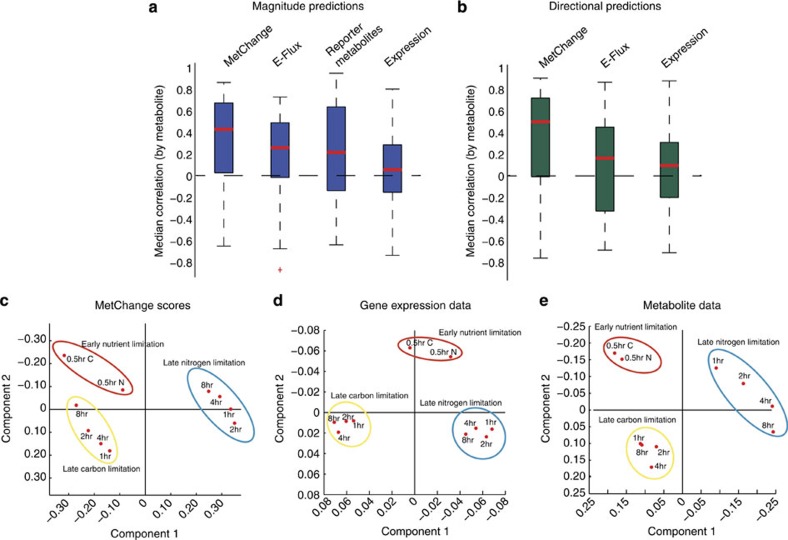
Analysis of MetChange analysis of matched transcriptomic/metabolomics data in *S. cerevisiae* under carbon and nitrogen starvation conditions using the *S. cerevisiae* metabolic network reconstruction iMM904. (**a**) Comparison of the median correlations of computational metabolite absolute magnitude perturbation predictions with experimental data for several existing methods of integrating gene expression data with a metabolic reconstruction. (**b**) The same comparison as in part **a**, but taking into account the direction of perturbation (the reporter metabolites method is not directional in its predictions, so both comparisons were made). Error bars are standard deviations. The MetChange algorithm performs favourably on this data set in both absolute magnitude and directional predictions. (**c**–**e**) Principle component analysis of the MetChange scores, gene expression data and metabolite data for the 60 metabolites that mapped to iMM904. It is seen that the functional association of data is conserved after transformation to MetChange scores, and the MetChange principle component clustering has topological similarity to both gene expression and metabolite data clustering. The number of biological replicates in the original study was 1.

To compare the MetChange method against existing approaches that predict a metabolic outcome based on gene expression data^18^,^24^, a published gene expression data from carbon and nitrogen starvation in *S. cerevisiae* was analysed^25^,^26^. A previously generated metabolic reconstruction of *S. cerevisiae*, iMM904 (ref. 27), was used to compute MetChange scores for each condition. Scores were compared with metabolomics data generated for the same conditions using the relative changes from the initial time point. A total of 60 metabolites across five time points for each carbon and nitrogen starvation were compared for both absolute (that is, magnitude) correlation and directional (that is, increased or decreased) correlation with MetChange scores. The MetChange algorithm compared favourably both with other network-based expression analysis methods and with the use of gene expression alone in predicting metabolic perturbations (Fig. 3). Reassuringly, k-means clustering and principle component analysis of MetChange scores, mapped metabolomics data, and mapped expression data suggest that MetChange scores maintain functional relationships between time points that are present in expression and metabolomics data (Fig. 3).

To further validate the metabolic perturbations predicted by the MetChange method using the CMap data set, we performed a number of high-throughput computational analyses comparing MetChange perturbations with drug response properties (Table 1). First, metabolite scores were compared with co-occurrences of drug-metabolite text terms in the PubMed database. A bootstrap analysis of Z-score permutations showed that PubMed drug-metabolite associations are recalled in a statistically significant manner (non-parametric perturbation *P*-value *P*<10^−3^ for 1,000 perturbations of MetChange scores). Second, it was found that known metabolic drug targets as found in the DrugBank database^28^,^29^ are significantly closer in reaction proximity to known highly perturbed metabolites than less perturbed metabolites for these drugs (median Wilcoxon rank sum *P*<1.65 × 10^−10^ for the highest scoring bin). Third, MetChange scores were found to be able to predict drug–protein literature co-associations within the PubMed database in a statistically significant manner (non-parametric perturbation *P*<0.01).

Finally, we validated perturbation predictions in targeted experimental measurements in MCF-7 cell culture under treatment by the drugs metformin, haloperidol and genistein. These drugs were chosen because of previous evidence suggesting significant metabolic perturbation by these drugs. Measured metabolites were chosen to have broad coverage of pathways and target highly perturbed pathways as predicted by MetChange. Drug concentrations were chosen based on previous *in vitro* studies using these drugs.

**Figure 4:**
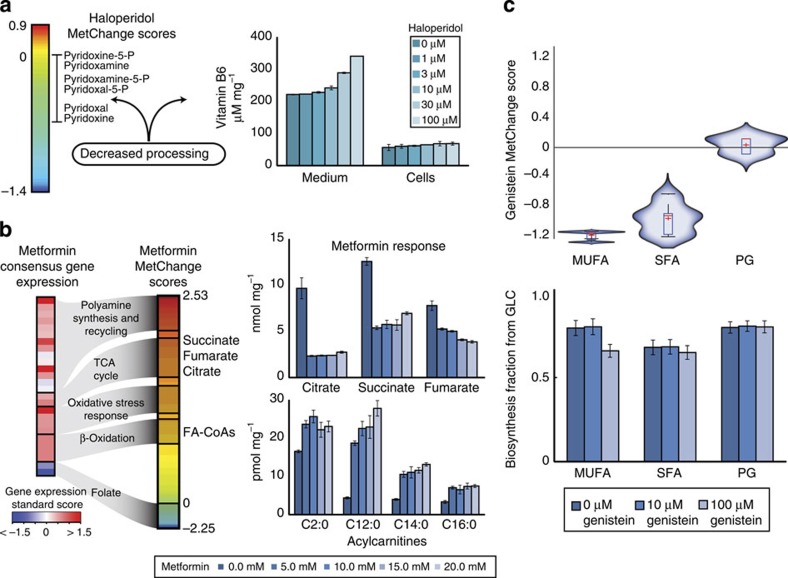
Validation in cell culture of drug-specific metabolic perturbations calculated from drug-induced transcription changes. (**a**) In the left panel, gene expression related to vitamin B_6_ metabolism is downregulated by haloperidol, suggesting decreased utilization of vitamin B_6_. In the right panel, vitamin B_6_ levels were measured by an enzymatic assay in media supernatant and lysates of MCF-7 cells, showing decreased utilization of the essential nutrient. (**b**) In the left panel, metformin gene expression perturbations are shown on the left, and resulting MetChange scores for related metabolites are shown on the right. The right panel shows response of tricarboxylic acid metabolites and acylcarnitines to metformin treatment. Metabolites show a large perturbation in the opposite direction of the observed transcriptional changes, validating the presence of a perturbation but indicating non-transcriptional control of metabolite levels in central metabolism as well. (**c**) In the upper panel, genistein is predicted to preferentially decrease production of mono-unsaturated fatty acids. The lower panel shows measurement of biosynthesis fraction of different fatty acid groups and precursors from glucose following genistein treatment measured by NMR spectroscopy, which validate modelling predictions. MUFA: Mono-unsaturated fatty acids. SFA: Saturated fatty acids. PG: Phosphoglycerate. Error bars represent standard deviations in all cases. Each experiment was performed in biological triplicate.

First, treatment with the antipsychotic haloperidol revealed decreased uptake of vitamin B6, consistent with the calculated decrease in the B6 processing pathway (Fig. 4a). Second, treatment with metformin, a 5′ AMP-activated protein kinase (AMPK) activator, showed significant perturbation of tricarboxylic acid cycle and fatty acid oxidation metabolites. The observed change was consistent with the large calculated perturbation but was in the opposite direction of the transcriptional change, indicating substantial non-transcriptional control of the metabolite levels (Fig. 4b). Additional calculated metformin-induced changes supported by previous results include: (i) a downregulation of folate metabolism consistent with reported folate deficiency in metformin-treated patients^30^, (ii) upregulated oxidative stress response consistent with reported lower reactive oxygen species levels^31^ and (iii) increased polyamine synthesis and recycling pathways that may result from shared use of organic cation transport (OCT) proteins between metformin^32^ and polyamines^33^. Third, treatment with genistein, an isoflavone with hypolipidemic effects^34^, experimentally showed a preferential reduction of the fraction of mono-unsaturated fatty acids synthesized from glucose that was consistent with predictions (Fig. 4c).

### Identification of *in vitro* disease-linked metabolic pathways

**Figure 5:**
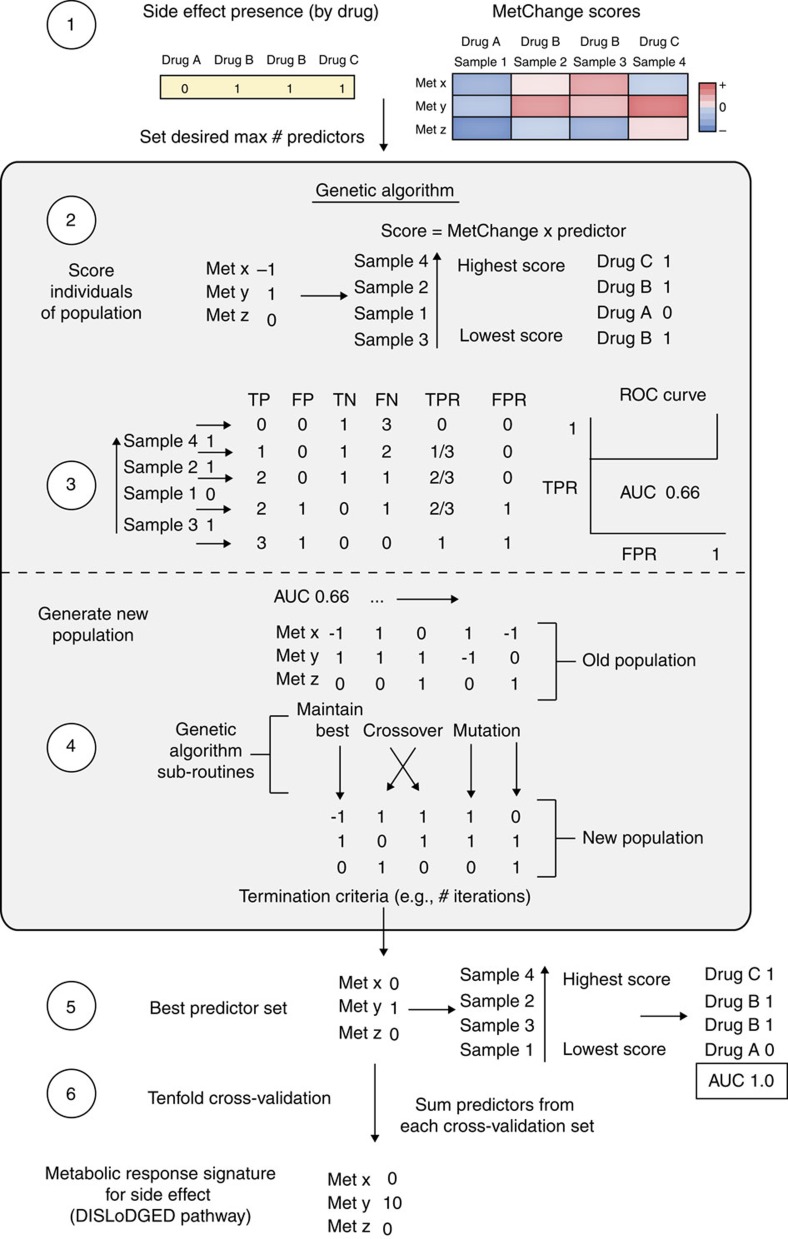
Description of the genetic algorithm used to identify metabolic signatures of side effects (DISLoDGED pathways). (1) Inputs to the algorithm are a set of response variables for each gene expression set (either MetChange scores or gene expression changes), a binary presence/absence vector for whether each sample was treated with a drug that has the side effect, and the desired maximum number of predictor variables desired. (2) At initiation, the genetic algorithm generates a ‘population' of random guesses at the predictor variables, termed ‘individuals', and assigns them either a value of −1, 0 or 1. For each individual, all gene expression samples are scored as the response variables (MetChange or gene expression changes) multiplied by the candidate signature. (3) Each gene expression sample is then ranked and a receiver operator characteristic (ROC) curve is generated and area under the curve (AUC) is calculated using the input presence/absence vector for the side effect or indication. The sample AUCs are the maximization objective of the genetic algorithm. (4) The genetic algorithm sub-routines are then used to generate a new population, biasing towards higher AUCs. Best solutions are maintained without modification, and lower scoring individuals are combined (‘crossed over') and modified (‘mutated') to search the solution space in a heuristic manner. The termination criteria are typically a number of generations without improvement; however, we applied a simple maximum time termination criteria, as obtaining a global optimum was not deemed essential to gain biological insight. (5) The signature yielding the highest prediction AUC is considered the best predictor set. In the example case, the resultant AUC is 1.0, a perfect predictor for the sample set. (6) To assess over-fitting and hence the predictive potential of the metabolic signature, tenfold cross-validation is performed by generating ten partitions of 90% of the data to train signatures and predict the remaining ten partitions of 10% of the data. To find signatures that have constant predictive power, the cross-validation signatures were summed, and high scoring metabolites were considered the conserved metabolic response signature (DISLoDGED pathway) for the side effect or indication. FPR, false positive rate; TPR, true positive rate; TP, true positive; TN, true negative; FP, false positive; FN, false negative.

Using MetChange scores generated from the CMap data set, we then identified the drug-induced metabolic gene expression changes that are most conserved among drugs with particular clinically described side effects. We used a machine learning approach to select discriminating metabolite production pathway perturbations from the set of MetChange-calculated pathway changes based upon side effect frequency data reported in the Side Effect Resource (SIDER) database^35^ (Figs 1b and 5). A total of 357 side effects across 1,417 gene expression profiles from CMap were analysed, based on the criterion that at least 30 expression sets per side effect were available. The cutoff in number of expressions sets was chosen rationally as a balance between the scale of the study and the robustness of the side effect signature obtained. Overall, 2,422 disease-linked drug-changed (termed DISLoDGED) metabolic pathways were identified with this analysis. These pathways are MetChange calculated metabolite production pathways that are over-represented in perturbations by drugs with particular side effects. DISLoDGED pathways are potential side effect to pathway relationships, hypothesizing that drug-induced perturbations away from metabolic homeostasis are involved in side effect pathogenesis. The calculated DISLoDGED pathways thus comprise a large set of omics-driven hypotheses of side effect pathogenic mechanisms (Supplementary Data 2). These associations were initially supported by automated co-association searches of PubMed abstracts to examine disease-linked nutrient deficiencies across all 357 side effects (Table 1). Downregulated DISLoDGED pathways were found to be marginally significantly predictive of deficiencies associated with corresponding specific diseases (permutation *P*=0.055), whereas upregulated pathways were not predictive (permutation *P*=0.39). We then sought to determine whether the metabolic expression changes induced by drug treatment were involved in the pathogenesis underlying drug side effects.

### Construction of a database of *in vivo* links between metabolism and disease

We assessed the relevance of DISLoDGED metabolic pathways to side effect pathogenesis through the use of a large body of clinical, biochemical and genetic literature on metabolism–disease relationships. We constructed a database of *in vivo* links found within the literature between metabolic function and disease, called the Metabolism Disease Database (MDDB), that consists of curated primary literature and existing databases (hosted at sbrg.ucsd.edu/mddb). Data collected through manual curation of the literature included disease-linked: (i) metabolic gene variants, (ii) physiological system-specific metabolism, (iii) metabolic pathways, (iv) chemical inhibitors of metabolism and (v) nutrient deficiencies and supplements. Data aggregated from existing databases included metabolic gene variants affecting disease incidences from a large genome-wide association study (GWAS) database^36^ and drug-metabolic enzyme target pairs from DrugBank^28^,^29^. Studies on disease models were treated as acceptable sources where clinical data were not available. The resulting database encompasses 357 side effects, over 280 non-drug inhibitors, 600 drug molecules, 37 nutrients and over 5,000 investigated metabolic pathway-disease-link associations. The database includes information related to both drug side effects and non-drug-induced pathologies, and we examined predictions in terms of each of these separately.

### Comparison of DISLoDGED pathways with side effect pathogenesis

We first examined whether DISLoDGED metabolic pathways contain genes with variants that alter clinical side effect susceptibility, according to the data in MDDB (Table 1). Causal gene variants are typically considered to affect either drug pharmacokinetics, which involves drug exposure, or drug pharmacodynamics, which involves the interaction of the drug with the body. The majority of identified gene variants affecting drug side effect incidence are involved in drug pharmacokinetics, because these genes historically have been simpler to identify as drug metabolism genes are largely known. However, we focused upon genes affecting pharmacodynamics, as these genes are more directly indicative of the mechanisms underlying pathogenesis. In MDDB, we identified nine side effect susceptibility gene variants that overlap directly with metabolism but are not involved in drug pharmacokinetics (Supplementary Note 1). We found that for each of the nine side effect susceptibility genes, at least one overlapping DISLoDGED pathway for this side effect had been identified through our analysis (Table 2). This overlap between DISLoDGED pathways and non-pharmacokinetic side effect susceptibility altering genes was highly significant (joint hypergeometric tests, *P*-value 2.2 × 10^−11^, see Supplementary Note 2 for calculation).

To support the relevance of this overlap, we assessed the nine overlapping DISLoDGED pathways in the context of additional known factors related to side effect pathogenesis (Table 2). We found that, in each case, the overlapping DISLoDGED pathway had established ties to the clinical pathogenesis of the side effect (Table 2, column 5). In the seven cases where the side effect pathology had been reported independently of the drug, alterations in the DISLoDGED pathway have been associated with the disease (Table 2, column 6). Critically, in each of the nine cases, we found that drugs causing the side effect had been reported to induce a perturbation in the DISLoDGED pathway *in vivo*, demonstrating that the *in vitro*-derived DISLoDGED pathways are similarly perturbed *in vivo* (Table 2, column 7). Furthermore, in seven of the nine cases, nutrient supplementation targeted at the DISLoDGED pathways have been shown to be effective in treating the drug side effect, whereas the remaining two cases are inconclusive due to reports of both positive and negative results (Table 2, column 8).

For example, among the 38 drugs within the CMap database reported in SIDER as causing increased risk of cardiac arrhythmias, we identified five DISLoDGED pathways (Supplementary Note 1). Three of these DISLoDGED pathways, which were a downregulation of oxidative pentose phosphate pathway and two related upregulations in nitrogen metabolism, overlap with genetic polymorphisms known to cause increased susceptibility to arrhythmias^37^,^38^,^39^ (Table 2). These pathways are known to have physiological ties to the pathogenesis of arrhythmias^40^,^41^ and have been shown to be perturbed *in vivo* by drugs causing arrhythmias^42^,^43^ (Table 2). Furthermore, nutrient supplementation targeted at these pathways has been found therapeutic in drug-induced arrhythmias^44^,^45^ (Table 2). The remaining examples are presented in Supplementary Note 1.

### Comparison of DISLoDGED pathways with general disease pathways

We further expanded the scope of validation beyond the nine available cases of non-pharmacokinetic genetic variants directly affecting side effect incidence. The MDDB database was used to determine whether the identified DISLoDGED pathways are conserved within *in vivo* data related to non-drug-induced pathologies as well, where a significantly larger body of literature exists than for side effect pathogenesis. This analysis hypothesizes that non-drug-induced pathogenesis and corresponding side effect pathogenesis share a common basis. Using the data collected, we assessed the calculated DISLoDGED pathways in terms of the five causal relationships in MDDB. Downregulated and upregulated DISLoDGED pathways were assessed independently to examine directional causal relationships. Approximately one-sixth of calculated DISLoDGED pathways were investigated for validation, and compared with an equal number of randomized predictions as a control. The next sections describe each causal link examined.

### Gene variants affecting disease incidence

We first sought to determine whether DISLoDGED pathways contain known disease-linked gene variants. To do this, we compared the metabolic subsystems into which disease-linked gene variants and DISLoDGED pathways occur. We analysed 239 metabolic disease-linked genes from MDDB and found an enrichment of disease-linked genetic variants among transporters (one-tailed hypergeometric *P*=0.0048) and inositol metabolism (one-tailed hypergeometric *P*=0.02), as well as a depletion of variants in central carbon metabolism (one-tailed hypergeometric *P*=0.035). We found that DISLoDGED pathways showed similar results, including an enrichment of downregulated DISLoDGED pathways in inositol metabolism (*P*=0.018) mirroring the enrichment found in disease-linked genetic variants, as well as enrichment of DISLoDGED pathways in non-central metabolism (Supplementary Table 1). These results indicate certain metabolic pathways may be inherently less robust to pathological perturbation.

### DISLoDGED pathway associations with disease physiology

**Figure 6:**
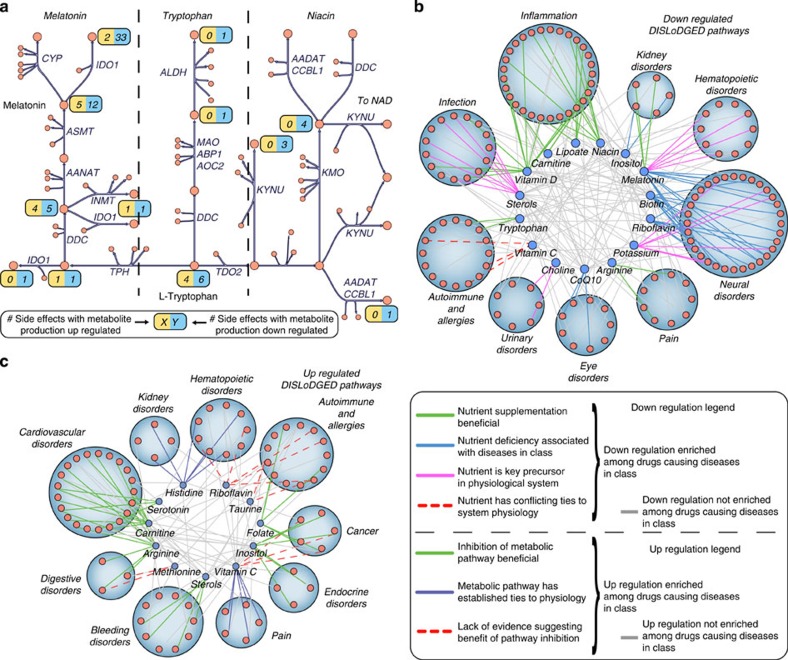
Maps of interactions between DISLoDGED pathways grouped by nutrient domain and corresponding side effects grouped by disease class. Only nutrients and disease classes with at least one marginally enriched nutrient-class interaction (hypergeometric *P*<0.1) are shown. (**a**) Using the metabolic network reconstruction Recon 1, side effect-specific metabolic perturbations (DISLoDGED pathways) are grouped into nutrient domains to enable comparison with existing disease-related genetic, clinical and pre-clinical data to assess the potential causality of observed perturbations. In this figure, the number of side effects with an upregulation in the production pathway for a metabolite is shown in yellow boxes, whereas blue boxes show the number of side effects with a downregulation in the production pathway for a metabolite. (**b**) Downregulated DISLoDGED pathways. Nutrient-disease class interactions indicating an enrichment of downregulations in drugs causing side effects within the class are coloured according to the legend. (**c**) Upregulated DISLoDGED pathways. Nutrient–disease class interactions indicating an enrichment of upregulations in drugs causing side effects within the class are coloured according to the legend. Many of the enriched interactions are consistent with known effects of nutrient/pathway perturbation on the corresponding disease classes and physiological systems.

Next, we examined whether DISLoDGED pathways are known to be essential to system physiology in a manner that could result in disease when perturbed. To compare DISLoDGED pathways to disease pathophysiology, side effects were grouped based on affected physiological systems, such as renal diseases or autoimmune complications. We first grouped DISLoDGED pathways by nearest nutrient for better coverage in the literature (Fig. 6a). DISLoDGED nutrient pathways preferentially altered by drugs causing side effects in specific physiological systems were then calculated. In 17 of the 18 cases of enrichments of downregulated DISLoDGED nutrient pathways within particular physiological systems, the downregulated pathways had directionally consistent links to the disease pathophysiology (Fig. 6b and Supplementary Data 2). For example, inositol metabolism downregulation was enriched among drugs with side effects affecting the kidney, including kidney failure. Supporting this relationship, the kidney is a primary site of inositol synthesis^46^, and inhibition of inositol transport has been reported to cause renal failure^47^.

Similarly, pathways that were upregulated by drugs affecting particular physiological systems also showed directionally consistent links to pathophysiology. In 9 out of 17 DISLoDGED nutrient pathways that were upregulated in particular physiological systems, inhibitors targeted at the upregulated pathway were established therapeutics within the disease class (Fig. 6c). For example, drugs causing cancer- or autoimmune-related side effects were enriched in upregulation of folate metabolism, and anti-folates are commonly used in treatment of diseases in both classes. Supporting the implications of this upregulation in side effect pathogenesis, studies have shown that folate supplementation is tied to increased incidence of both cancer^48^ and childhood asthma^49^.

### Altered activity of DISLoDGED pathways in disease

**Figure 7:**
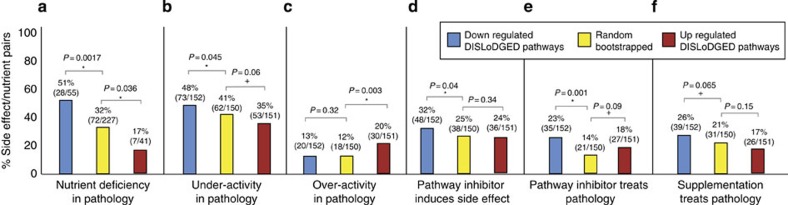
Multiple lines of clinical evidence in MDDB implicate a causal role for drug-induced metabolic transcription changes in side effect pathogenesis. (**a**,**b**) Downregulated DISLoDGED pathways are found to be enriched in nutrient or pathway deficiencies associated with the corresponding pathology. (**c**) Upregulated DISLoDGED pathways are found to be enriched in consistently over-active pathways associated with corresponding pathologies. (**d**) Inhibitors targeted at DISLoDGED metabolic pathways are more likely to effective treatments in corresponding pathologies. (**e**) Metabolic inhibitors targeted at downregulated DISLoDGED pathways are significantly more likely to cause corresponding side effects. (**f**) Nutrient supplementation targeted at downregulated DISLoDGED pathways was found to preferentially alleviate the corresponding pathology. *P*-values are defined for one-tailed binomial tests based on the level of the treated sample relative to the control (random). Sample sizes were chosen *a priori* based on feasibility of data collection, without an expectation for a particular effect size. Symbols: * indicates significant or *α*<0.05, +indicates marginally significant or *α*<0.1.

We then sought to determine whether DISLoDGED pathways are significantly over- or under-active in corresponding clinical disease and disease models, based on several metrics in the MDDB. To perform this analysis, DISLoDGED metabolic pathways were associated with dietary nutrients nearest in the metabolic network. In an initial analysis of 323 DISLoDGED nutrient pathways, downregulated DISLoDGED pathways were found to be significantly enriched in disease-associated nutrient deficiencies (59% enrichment, binomial *P*-value 0.0017), whereas upregulated DISLoDGED pathways were depleted in disease-associated nutrient deficiencies (47% depletion, binomial *P*-value 0.036; Fig. 7a).

These results were confirmed in an independent set of 453 investigated DISLoDGED pathways added to MDDB. Downregulated DISLoDGED pathways were more likely to have a causal downregulation associated with the corresponding pathology (17% enrichment of downregulation, binomial one-tailed *P*=0.045; Fig. 7b), whereas upregulated DISLoDGED pathways were significantly predictive of consistently over-active pathways tied to corresponding pathologies (67% enrichment of over-activity, binomial one-tailed *P*=0.003; Fig. 7c).

### Effect of targeted inhibition of DISLoDGED metabolic pathways

Next, we analysed *in vivo* data on the effect of non-drug chemical inhibitors targeted at calculated DISLoDGED pathways. A causal relationship would be indicated by (i) inhibitors targeted at downregulated DISLoDGED pathways reproducing the clinical side effect and (ii) inhibitors targeted at upregulated DISLoDGED pathways treating the clinical disease. We found that metabolic inhibitors targeting downregulated DISLoDGED pathways specifically were significantly more likely to cause corresponding side effects (28% enrichment, binomial one-tailed *P*=0.04; Fig. 7d). Also, we found that both downregulated and upregulated DISLoDGED pathways were more likely to be known targets for effective metabolic inhibition to treat corresponding diseases, indicative of imperfect directionality of predictions (64% enrichment, binomial one-tailed *P*=0.0015 and 29% enrichment, binomial one-tailed=0.09, respectively; Fig. 7e).

### Effect of supplementation targeted at DISLoDGED pathways

Finally, we compared calculated DISLoDGED pathways grouped by nearest nutrient with clinical therapeutic nutrient supplementation data (Fig. 6a). We sought to determine whether downregulated DISLoDGED pathways might be targets for nutrient supplementation as a disease therapy. We observed that predicted downregulated DISLoDGED pathways were preferentially targets of nutrient supplementation to alleviate corresponding pathologies (24% enrichment, binomial one-tailed *P*=0.065; Fig. 7e), whereas overexpressed pathways were preferentially depleted as effective nutrient supplements (19% depletion, binomial *P*=0.14), although the relationships did not reach statistical significance for given sample sizes available.

## Discussion

The systems biology-based workflow developed in this study predicts side effect-linked dysregulated metabolic pathways (termed DISLoDGED pathways) from drug treatment of human cells in culture. We contextualize numerous independent data types that together support a key role of drug-induced non-pharmacokinetic metabolic dysregulation in side effect pathogenesis. Through the construction of a comprehensive resource on metabolic involvement in disease, we corroborate the predictions made using five independent clinical, genetic and biochemical bodies of the literature. These results provide understanding of the mechanisms underlying side effect pathogenesis, which have remained largely opaque despite recent progress identifying causal protein-binding events and genetic susceptibility factors.

The work presented relied upon the development of a systems biology workflow to identify side effect-linked features and validate the relevance of these features using historical *in vivo* data. The use of systems biology approaches to study drug side effects has become an active field in recent years. Previous studies have predicted drug–protein-binding events responsible for side effects^9^,^50^, effective combinations of drugs to minimize side effects^51^, and drug mechanisms of action underlying side effect pathogenesis^12^,^52^. Still, uncovering how causal binding events result in disease is an essential and largely unanswered question, as identified pathogenic mechanisms can potentially be used to design therapies to circumvent drug side effects. The present work we believe is the first to validate omics-driven predictions of post-binding mechanisms underlying side pathogenesis using clinical data at a large scale, which was empowered by the generation of a new database through manual curation of the literature.

The workflow presented is highly dependent upon large amounts of gene expression data obtained under treatment by diverse drugs to filter out perturbations due to factors other than common side effects. Normalization of data across multiple studies and platforms is a substantial issue. Thus, data generated within a single study is ideal, but such large studies are rare. For this reason, the potential to extend the workflow to new data types, for example, *in vivo* gene expression data, may be limited. Owing to the use of *in vitro* data, corroboration against *in vivo* data appears critical. Further deployment of the presented workflow may hinge upon expansion of curated disease data necessary to corroborate the clinical relevance of *in vitro* drug treatment features. Furthermore, the definition of pathways used to integrate disparate data types has yet to be concretely established and may be an area for further workflow optimization.

Owing to the ubiquity of gene set enrichment analysis (GSEA)^53^ in pathway-based analysis of gene expression, a discussion on the differences between the MetChange algorithm and GSEA is warranted. Both methods attempt to aggregate ‘signal' in gene expression data along pathway definitions to increase the interpretability of the data and decrease the effect of noise. GSEA uses a variety of pathways, including manually curated metabolic pathways, and results are typically *P*-values of a Kolmogorov–Smirnov test for the likelihood that the cumulative distribution of expressions of genes in each pathway have not changed between conditions. MetChange defines a different production pathway for each metabolite in the network, based on calculation of functional states of the metabolic network, and scores for each metabolite how gene expression along this production pathway has changed between conditions. As a result, MetChange has the potential to give finer resolution results, as its results are defined at the level of individual metabolite scores rather than pathway scores. However, the overall performance of the methods are difficult compared due to the fundamental difference in resolution of outputs.

In the comparison between MetChange and comparable methods (Fig. 3), it is apparent that, on the data set used, output correlations of none of the methods with measured metabolite perturbations in yeast were particularly high. However, given that this analysis is possibly using these methods out of their intended use cases, no presumptions should be made regarding the general usefulness of any of these methods. Rather, this may highlight the difficulty in establishing standards by which to compare pathway analysis algorithms with statistical rigor. In this study, we performed the analysis primarily to provide some context for the relative performance of the MetChange algorithm at one specific task relevant to predicting metabolic changes occurring within the cell. We note that a recent method to predict metabolomics changes from gene expression has reported statistically significant correlations on three other yeast data sets^54^, including values that exceed those reported in Fig. 3.

There are at least two obvious potential improvements that could be made to the workflow used in this work. First, as the cell lines within the CMap are within the NCI60 cell line panel, and expression, growth and most recently exometabolomic^12^ data have been measured for these lines, cell-specific metabolic models could be used in place of a global model. Second, the human metabolic network reconstruction has been updated with the publication of Recon 2 (ref. 55), and thus improvements might be made from the increased scope of the model. We evaluated the potential for improvements using these changes by constructing cell-specific models for the MCF-7, HL-60 and PC-3 cell lines based on Recon 2 and running the MetChange algorithm on randomized simulated expression data drawn from an empirical distribution of MAS5.0 normalized *P*-values from the CMap data used in this study. Briefly, between cell-specific Recon 2 models, error was between 10 and 25% across replicates when given the same metabolite uptake constraints. The largest difference observed was by enforcing measured metabolite uptake constraints. Differences in MetChange scores between models constrained and unconstrained by metabolite uptakes were around 70%, indicating that significantly constraining the flux state of the model alters MetChange scores more so than topological differences. Thus, further improvements in prediction accuracy might be seen by accounting for the baseline metabolic differences between cell lines.

One interesting outcome of the work was that, although previously identified genetic susceptibility factors in the literature are dominated by genes involved in pharmacokinetics, we observe overlap of drug-induced metabolic changes with nine genes that affect pharmacodynamics. This may suggest that such non-pharmacokinetic genes may play a larger role in side effect pathogenesis than currently appreciated. We did, however, see some alteration of pharmacokinetic genes in a few cases as well (Supplementary Note 3). In addition, DISLoDGED pathways overlapped with clinical disease-linked pathways for both drug-treated and drug-independent studies, suggesting a common basis in pathogenesis. Furthermore, our results suggest that targeted nutrient supplementation may be a relatively simple and inexpensive path to broadly reduce side effect incidence. The impact of drugs on patient metabolic status is thought to be an underappreciated but important aspect of drug response^56^, and this work further suggests this interaction is worthy of significant investigation. Patient attrition may be reduced through effective nutrient supplement to drug pairing during the development process.

A natural question that may arise is whether certain pathways or side effect disease classes are more successfully predicted by the method used in this work than others. A sensitivity analysis shows that the method appears to be fairly robust in being able to predict DISLoDGED pathways in various areas of the metabolic network and across disease classes. Restricting to at least five pairs investigated for both the down-regulated DISLoDGED pathways and random pairs, deficiencies of nine nutrients (polyunsaturated fatty acids, coenzyme Q10, melatonin, niacin, riboflavin, steroids, thiamine, vitamin A and vitamin D) had disease associations better predicted by down-regulated DISLoDGED pathways compared with random, whereas one (choline) did not. Similarly, in side effects with at least two pairs investigated in both random and real, seven side effect/nutrient deficiency relationships were better predicted by down-regulated DISLoDGED pathways (dyspepsia, epilepsy, hyperlipidemia, interstitial nephritis, tardive dyskinesia, testicular atrophy, and thrombocytopenia), whereas only two were better predicted by random nutrients (ecchymosis and tendonitis). These types of sensitivity analyses on predictive capability for particular nutrients and side effects are vulnerable to error because of small sample size but corroborate overall results that dysregulated DISLoDGED pathways are predictive of metabolic pathways associated with corresponding pathologies.

Although challenges remain, the ability to observe perturbations important to *in vivo* side effect susceptibility within *in vitro* data suggests that early-stage drug screening to identify and manage side effect risk may become possible, analogous to other ‘disease in a dish' efforts^57^. Notably, high use drugs such as statins and antipsychotics, where patient populations are large enough for statistical analysis of rare side effect events, dominate cases of side effects where genetic components have been identified. However, if vulnerable pathways can be identified through analysis of *in vitro* data, it may become easier to identify susceptibility factors for more rare disease classes as well. This workflow is currently limited to cases in which gene expression alteration underlies side effect pathogenesis, which is an undefined subset of all side effects. The results presented show the utility of integrating large, standardized data sets, such as the CMap, with clinical data types such as side effect incidence, genetic studies and disease–nutrient associations, in the context of a highly annotated network with clear functional connections. Such integration of disparate data sources is a key challenge in many areas of the life sciences today.

## Methods

### Overview of computational approach

As described schematically in Fig. 1, with more detailed methods diagrams in Figs 2 and 5, we employed a combination of constraint-based modelling and machine learning to look for metabolic gene expression perturbations that are conserved in adverse drug reactions. The first step is the analysis of drug-specific metabolic perturbations using the constraint-based MetChange algorithm described below and comparison of these perturbations to drug-specific response properties. We then combined MetChange scores based on side effects and used an area under the Receiver Operating Characteristic curve (AUC)-maximizing classification genetic algorithm described below to determine small subsets of metabolic changes highly conserved in certain side effects. We note in general that the fields of constraint-based modelling and machine learning have developed a wide variety of methods that perform similar tasks. We compare our method with several other constraint-based methods and with gene expression data alone, as described in the Results. We also qualitatively contrast our method with GSEA^53^ in the Discussion. In general, performance of machine learning methods largely depends on the particular problem. We note that in cases of rare events, such that only a small fraction of samples have a property, AUC (or rank) maximizing algorithms have been shown to perform particularly well. In addition, we choose to place a hard constraint on the number of variables rather than using a traditional SVM with an L2-norm approach, for example. This was done to maximize interpretability of the resulting signatures, which was critical for later comparison of DISLoDGED pathways with pathology deficiency relationships and disease-linked and side effect-linked genetic susceptibilities. Thus, although we do not discount that other methods may exhibit better performance by certain standards, we chose our approach to meet the specific requirements of our problem, as is typical in the field. Full description of methods is below and a MATLAB implementation is provided (see Supplementary Software).

### CMap data processing and integration

Gene expression data for the AffyMetrix HT Human Genome U133A platform were obtained from the CMap database^22^ for MCF-7, PC-3 and HL-60 cell lines. Data were MAS5.0-normalized with the BioConductor package^58^. Human Entrez Gene identifiers associated with probes were used to map detection *P*-values to their corresponding reactions based on the Boolean gene–protein–reaction associations as was previously described^59^. Reactions that were not associated with a gene were assigned a *P*-value of 0. Metabolic exchange was set to an exchange value of −1 for DMEM (MCF-7) or RPMI (PC-3 and HL-60) media metabolites. In cases where no metabolite production was possible with open constraints, the metabolite was removed from further analysis. For the cases of *in vitro* experimental validation under genistein treatment, for which data was generated for the fraction of metabolites generated from glucose, the MetChange algorithm was run using glucose as the sole carbon source to enable direct comparison with the data.

### The MetChange algorithm

The Metabolite-Centered Hotspots of Altered Network Gene Expression (MetChange) algorithm was used to analyse differential quantitative gene expression profiles in the context of a genome-scale metabolic network^19^. This algorithm is built upon the Gene Inactivity Moderated by Metabolism and Expression (GIMME) algorithm previously developed to build context-specific metabolic networks based on gene expression data^59^,^60^.

The MetChange algorithm defines a consistency metric of an expression profile with optimally producing each metabolite in the metabolic network. For each metabolite, a sink reaction is created and flux through the sink reaction is maximized using flux balance analysis:


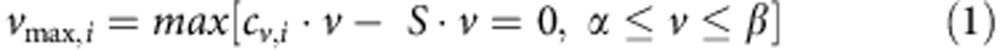


where ***S*** is the stoichiometric matrix, ***v*** is the reaction flux vector, ***α*** and ***β*** are vectors for the lower and upper bounds of the reactions and **c**_***v,i***_ corresponds to the imposed reaction objective for each *i*th sink reaction. This results in a set of optimal production fluxes for each network metabolite.

Each reaction is then weighted by a detection *P*-value from mapped expression data to solve a second optimization problem. To obtain metabolite production ‘consistency' scores, *x*_*i*_, for each *i*th metabolite, scores were generated by setting the lower bound of the metabolite sink reaction to its maximal production flux, *v*_max*,i*_, and minimizing the inner product of the reaction detection *P*-values:


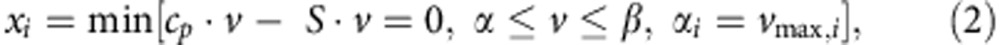


where mapped detection *P*-values ***p*** were stored in a weighting vector ***c***_***p***_, and each vector component *c*_*p,i*_=*p*_*i*_ mapped to each *i*th reaction. This optimization is the GIMME algorithm-like step of the MetChange algorithm. Each *i*th component in ***c***_***p***_ serve as a cue for whether a reaction is detected or absent (that is, a lower *P*-value indicates a reaction is more likely to be present). Hence, this second optimization determines: (i) a flux distribution which maximizes the production of a metabolite and (ii) generates a weighted flux distribution score defining consistency of the production of that metabolite with expression data. These scores cannot be directly compared across metabolites because of the fact that each metabolite has a different network flux state at maximal production. Thus, we compared metabolite production consistency scores between treated and control samples with a standardized score:


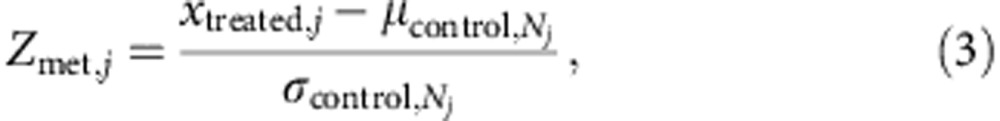


A MATLAB implementation of the MetChange algorithm is provided in the Supplementary Software File.

### Analysis of expression data for yeast under carbon and nitrogen starvation

Metabolite concentration^25^ and gene expression^26^ data for *S. cerevisiae* were mapped to a genome-scale yeast metabolic reconstruction, iMM904 (ref. 26). For all methods, scores were compared to log-2 metabolite concentration changes relative to the initial time point for carbon or nitrogen data. Spearman correlations were calculated to avoid biases due to variable score distributions among the different methods. K-means clustering analysis was performed using 100 replicates. In all cases, the same clusters were obtained in repeated runs.

For the MetChange algorithm, reported expression values were used to generate reaction presence/absence *P*-values. Expression values were ranked, and based upon the apparent distribution, a value corresponding to the bottom second percentile of expression was used to define the noise level. Expression values below this threshold were assigned a *P*-value of 1 as ‘not present.' The *P*-values below the threshold were then inverted across the threshold to generate a symmetric distribution. The mean and standard deviation of this approximated noise distribution were used to generate significance scores for the remaining expression values using a Z-test. These *P*-values were then mapped to reactions based on model-defined gene–reaction relationships. When multiple probes were assigned the same reaction, the minimum value was used. The MetChange algorithm was then applied using these mapped reaction presence/absence *P*-values. As the data were longitudinal in time and multiple controls were not available, log-2 scores were calculated with respect to the initial time point, rather than taking standard scores.

Reporter metabolite analysis was then implemented. *P*-values for the significance in expression change were used as inputs to the reporter metabolite analysis. Each expression value was first standardized across conditions and then *P*-values were calculated using a standard Z-test for each gene across condition. 10,000 permutations of the data were used to generate the background levels of perturbation.

In addition, the E-Flux method was implemented^18^, with metabolite sinks set as separate objective reactions similar to the MetChange algorithm for comparison. Optimal flux through each metabolite sink reaction was then calculated using flux balance analysis for each gene expression data set, and log-2 scores compared with the initial time point for each carbon and nitrogen data set were calculated.

For a direct comparison with gene expression level cues, we implemented the following approach. First, standardized scores of expression level changes were calculated with respect to all gene expression data sets for the particular perturbation (carbon or nitrogen starvation). The scores were mapped to their respective reactions according to the gene–reaction association in the metabolic network. We then assessed whether absolute changes in expression levels are predictive of the magnitude of metabolite change by adding the standardized reaction scores, weighted by the absolute stoichiometric coefficients. We then assessed whether higher gene expression for a reaction was indicative of a higher product concentration and lower reactant concentration. The mapped reaction score was added to the score for all metabolites in the reaction, multiplied by the signed stoichiometric coefficient for each metabolite.

### Analysis of metabolic response phenotypes across drugs

The Recon 1 metabolic network^19^ was first converted into an irreversible network, such that each reaction proceeds only forward, and reactions that can proceed in multiple directions are split into two forward-proceeding reactions. The MetChange algorithm was run using gene expression presence/absence MAS5.0 *P*-values from the CMap database build 02 (ref. 22). When multiple controls were present, a standard score was generated. When a treated sample was from a batch with a single control, the mean and standard deviation of all control samples was used instead.

Cell line standard scores were then generated in the following manner. First, for each cell line, the median scores of all samples for each drug were found and used as the cell line-specific response. Then, to simplify compartment-specific scores to a general metabolite response, cytosolic metabolite scores were taken when available. If no cytosolic metabolite existed, the median of scores across all compartments was taken as the metabolite score. Finally, a standard score across all drugs was calculated for each cell line. Consensus drug perturbations across cell lines were calculated by averaging cell-specific MetChange scores and standardizing across all drugs.

### PubMed querying of drug associations

To identify drug-metabolite associations in the literature in an automated manner, PubMed/MEDLINE records were downloaded from the National Library of Medicine and abstracts were parsed to raw text. Chemical entities were tagged using Reflect^61^. A training set of 44 abstracts describing true drug-metabolite correlations (positive set) and 150 abstracts with other chemical entities (negative set) were used to train a Bayesian network to recognize abstracts that mention the causative relationship between an administrated drug and the presence of a certain metabolite. A second Bayesian network was trained to recognize sentences within the abstracts that refer to metabolites from those that refer to other chemical entities. For each drug-metabolite co-occurrence the two Bayesian networks were used to assign a posterior for abstract occurrence and a posterior for sentence occurrence and the joint probability of the two posteriors was used as the drug–metabolite score. The evaluation of the output is provided in Supplementary Data 1. The text-mining output was evaluated using a random sample of 100 pairs. The abstracts that describe each sampled pair were manually checked and identified as true or false positives. The score cutoff was set to 0.33, which provides an 82% true positive rate and 70% accuracy.

Text querying on side effect–metabolite, protein–metabolite and drug–protein co-occurrences were performed using the Entrez Programming Utilities (NCBI) using a simple ‘AND' specification. Side effects were obtained from the SIDER database^35^. Signalling protein lists were obtained from various publically hosted resources including the International Union of Basic and Clinical Pharmacology database (http://www.iuphar.org/). Metabolite common names were obtained from the Recon 1 network reconstruction^19^. All associations are included in Supplementary Data 1.

### Determining metabolite network distance from drug perturbation

To calculate network distance from metabolites, the DrugBank database^28^,^29^ was downloaded and cross referenced with drugs from CMap. A total of 134 drugs present in CMap have targets in Recon 1 reported in DrugBank. Across the three cell lines (HL60, MCF7, PC3) and multiple drug concentration ranges, there were a total of 611 expression profiles of drug-perturbed states. The median metabolite network distance of each metabolite was calculated for each of the 134 drugs, ignoring metabolites with reaction connectivities greater than 30, such as cofactors, protons and water.

MetChange scores were compared against expression data, randomized data and reporter metabolite analysis results. Data and scores were pooled into bins based on their standard scores. For each bin, the average of the median network distance of metabolites to metabolites of known drug-targeted enzymes was calculated for comparison. Metabolites predicted from the expression data set were determined by taking the highest expression change among reactions involving the metabolite and binning accordingly. The random data set was generated by permuting the expression data set 1,000 times and calculating the average bin value across all 1,000 sets. The reporter metabolite data set was generated as described in the original publication^24^ using 10,000 permutations to generate background perturbation levels.

### Analysis of drug-signal protein signatures

Drug–protein and protein–metabolite literature co-associations were found as described. Associations were binary based on the presence or absence of known literature association. A receiver operating characteristic (ROC) curve was generated for the ability of MetChange scores to indirectly predict drug–protein association. MetChange scores for all three cell lines (not averaged) were used together. At increasing thresholds from 0 to effectively infinity, metabolites with absolute MetChange scores greater than the threshold were scored as significantly changed for each drug. Proteins associated with perturbed metabolites were then determined using the protein–metabolite literature co-associations. These proteins were used as guesses for true drug–protein literature associations for the drug corresponding to the sample, and the true positive rate and false positive rate were calculated. Varying the threshold from 0 to effectively infinity then generates the ROC curve. To assess statistical significance of the resulting AUC, 1,000 permutations of MetChange scores were analysed in the same way and a non-parametric rank test was conducted on the resulting AUCs.

### Determination and analysis of drug side effect signatures

Drug side effects were taken from SIDER database^33^ for available drugs overlapping with the CMap database. The SIDER database contains minimum and maximum occurrence frequencies for a number of both treated and control studies for each drug–side effect pair. Side effect frequencies were processed in the following manner. The mean of the minimum and maximum frequency was calculated for each study, and then the median of frequencies from all studies was found for both treated and placebo studies. The difference between treated and placebo occurrence frequency was then calculated. If placebos were not available, the treated frequency was used. These frequencies were then mapped to all expression samples from CMap corresponding to the appropriate drugs. A minimum of 30 expression sets for a side effect were required for inclusion in the analysis. A total of 357 side effects were analysed for 850 expression sets corresponding to 334 drugs.

A genetic algorithm was then implemented, termed SiderFinder (Fig. 5). The matrix of MetChange scores for the 850 expression sets was input as well as the corresponding side effect frequencies for a particular side effect. A maximum number of predictor metabolites was set to 20 metabolites. A set of 125 candidate solutions were generated, assigning values of −1, 0 or 1 to each metabolite to indicate negative, no or positive prediction of a high MetChange metabolite score on occurrence of side effect. Each expression set was then scored for the side effect for each individual by multiplying the predictor set for the individual by the MetChange scores for the expression set. These side effect scores were ranked and a pseudo-ROC curve was generated by comparison of scores with the side effect frequencies for the current side effect. At each threshold, expression sets with a side effect score greater than the threshold were called as having the side effect. To weigh more heavily affecting samples with higher side effect frequency, the base 10 logarithm of each side effect frequency was taken and adjusted such that the lowest non-zero frequency had a value of 1, each order of magnitude greater in frequency is a unit greater, and all zero frequency side effects remain zero. An ROC curve was then calculated with true positive hits being assigned the value of the adjusted side effect frequency, with no effect to false positive, true negative and false negative values. The number of true values was taken to be the sum of the adjusted frequency vector so the AUC of the pseudo-ROC still spans 0 to 1. The AUC of this curve was then used as the objective function to maximize in the genetic algorithm. Tenfold cross-validation was performed, and perturbations that appeared cumulatively in at least four of the ten sets we selected. Using the genetic algorithm directly on gene expression data achieved similar classification performance (results not shown), but metabolite scores were chosen as the variables due to their previously established performance in predicting drug-specific metabolic perturbations.

Genetic algorithm creation, mutation and crossover parameters were used as implemented in the OptGene function of the COBRA Toolbox 2.0 (ref. 62). The genetic algorithm was solved using the Global Optimization Toolbox in MATLAB (MathWorks). A MATLAB implementation is provided.

### Co-association analysis of drug side effect signatures

Statistical analysis of literature co-associations of side effects with side effect metabolite signatures was performed with (i) a non-parametric permutation test on the drug side effect metabolite signatures against 1,000 permutations of the signatures for the AUC of predicting presence/absence of literature side effect/metabolite co-association in PubMed abstracts and (ii) a hypergeometric test for the enrichment of literature association among side effect/metabolite pairs in predicted signatures.

### Construction of MDDB

To enable statistical analysis of predicted DISLoDGED pathways, disease–nutrient pairs were randomly selected from observed downregulations, observed upregulations and random associations chosen by resampling the former two lists through bootstrapping. These disease–nutrient pairs were then evaluated for existence of established relationships using manual literature searches while blind to the origin of the pair to prevent investigator bias. The list of collected metabolism–disease relationships is not yet comprehensive because of the scope of the effort, but instead relationships were investigated in a targeted manner.

Data collection for the database covered literature up to and including May 2013.

### Pathway analysis

We curated a database of 1,394 distinct GWAS publications^34^ and extracted 239 distinct disease-associated metabolic genes that overlapped with the 1,496 genes in Recon 1. We then assigned pathways to each disease-associated metabolic gene as well as to each DISLoDGED pathway calculated in our analysis (Supplementary Data 1). Disease-associated genes were assigned pathways based on the previously assigned pathway of corresponding reaction assigned in Recon 1. DISLoDGED pathways (which are metabolite centred) were associated with disease-linked pathways by determining the most frequent pathway among all reactions in which a metabolite takes part. To assess enrichment, hypergeometric tests were performed to determine whether the observed coverage of metabolites or genes was enriched or depleted in particular pathways, controlling for multiple hypothesis testing.

### Side effect pathophysiology classifications

To assess whether common metabolic perturbations were observed among related diseases, we manually grouped side effects by pathophysiological disease class. Enrichment was assessed with the hypergeometric test, at a significance level of *α*=0.1. Enrichment where there was only a single disease within the class was discarded, as were in conflicting cases (in which a nutrient was downregulated in certain diseases in the class and upregulated in others).

### Side effect-linked gene polymorphism search

We attempted to identify all cases of genetic basis for side effect incidence reported in the literature, including GWAS and targeted genetic studies. To be eligible for comparison, we required that the side effect be a near or exact match and the metabolic pathway of the susceptibility gene be within the scope of our model. For example, immune-related genes were excluded due to nonspecific metabolic association, whereas G protein–coupled receptors known be regulated by particular metabolic pathways were included. We generally excluded genes related to drug pharmacokinetics, including drug metabolism and transport, as the effects of polymorphisms on susceptibility are generally nonspecific to the pathology. We also required that the pathology be manifested in nucleated cells (that is, excluding red blood cell pathologies), as gene expression changes are assumed to be irrelevant to pathologies of enucleated cells. Based on these criteria, well over 20 studies were excluded, whereas 9 genetic susceptibilities spanning 6 side effects were valid for comparison with predictions. We also mention two cases in which pharmacokinetic genes do overlap with conserved side effect-linked gene perturbations, suggesting possible interactions between pharmacokinetic and gene expression effects of drug perturbation. Lists of included and excluded studies are found in Supplementary Notes 3 and 4, respectively.

### Nutrient deficiency literature search

To populate MDDB, we searched the literature for associations between the pathology of the side effect and deficiency relationships between the closest nutrient to the metabolic perturbation and the occurrence of the pathology of the side effect. In an automated search, PubMed abstracts were searched for a number of side effect and nutrient synonyms along with a list of deficiency synonyms (Supplementary Data 2). Statistical significance of enrichment of co-association PubMed abstract hits (presence/absence) among downregulated and upregulated nutrient pathways was assessed through a dual permutation analysis. For each perturbed side effect–nutrient pair, 1,000 permuted pairs were generated by first randomly selecting the presence/absence result for a random nutrient with the same side effect, and then randomly selecting the presence/absence result for a random side effect with the same nutrient, then averaging the result. Then, the number of permutations with total presence calls greater than the true observation was counted and divided by the total permutations, as is typical in permutation tests.

In the manual alteration search, a number of possible nutrient–disease relationships were identified, such as an inhibitor of the metabolic pathway causing the side effect, an inhibitor of the metabolic pathway curing the side effect and so on (Supplementary Data 2). Then, search terms were generated using synonyms, and PubMed was searched. ‘Results were then filtered such that each nutrient–side effect pair was assigned as ‘upregulated activity associated with the disease', ‘downregulated activity associated with the disease', ‘no associated with the disease' or ‘conflicting associated with the disease'. Inconsistent results were assigned as conflicting and were excluded from further analysis.' Significance of enrichments of particular relationships among upregulated and downregulated pathways were then evaluated with the hypergeometric test.

In the manual deficiency search, deficiency relationships were defined such that the DISLoDGED nutrient pathway could meet any of three possible criteria to be considered a ‘hit.' First, the deficiency of the nutrient could be known to be associated with the occurrence of the side effect pathology. Second, supplementation with the nutrient is known to alleviate the side effect pathology. Third, a physiological dysregulation of the pathway is known to be associated with the side effect pathology. PubMed and Google Scholar were both used for this study. Patents were not accepted as valid references, unless associated with a peer-reviewed publication.

To determine whether DISLoDGED pathways are significantly predictive of side effect/nutrient deficiency relationships, we generated a list of ‘random' side effect/nutrient pairs for comparison with DISLoDGED pathway–disease pairs through resampling of the pairs. Kolmogorov–Smirnov tests were used to ensure the distributions were not significantly different between DISLoDGED pathways and random side effect–nutrient pairs in terms of the frequency of occurrence of nutrients, as resampling should guarantee.

We then performed the nutrient deficiency literature search in two phases. The first was blinded and the second was an expansion and additional curation of the blinded study. The initial study was blinded to ensure that there was no selection bias in the search process. Random pairs were mixed with DISLoDGED pathways, both upregulations and downregulations, and information on their origin was removed. The literature searches for deficiency relationships were then conducted by investigators not involved in the generation of the distribution and thus were unaware of the treatments or treatment distributions. Statistical tests were only performed on the blinded literature results. The initial blinded list was then expanded to examine additional DISLoDGED pathways and curated to ensure consistent criteria between investigators. Although the curated list is unblinded, we have a greater confidence in this list for research purposes due to the consistent criteria and expanded list of investigated relationships.

### Overview of experimental validation of MetChange results

Drug-induced metabolic perturbations calculated using the MetChange algorithm were validated using *in vitro* experiments in the MCF-7 cell line for three drugs: metformin, genistein and haloperidol.

### Sample preparation

Human MCF-7 breast carcinoma cells (American Type Culture Collection, HTB-22) were maintained in supplemented DMEM media (CellGro Mediatech, 10013CV) with 10% (v/v) fetal bovine serum (Hyclone, SH3039603), 1% (v/v) antibiotic/antimycotic solution (Omega Scientific, PS-20), 1% (v/v) non-essential amino acids (Hyclone, SH3023801), 1% (v/v) MEM vitamins (CellGro Mediatech, 25020CI), 1 mM L-glutamine (CellGro Mediatech, 25005CI). 4 × 10^6^ cells were seeded in supplemented MEM medium (CellGro Mediatech, 15010CV) into 150 mm dishes. For labelling with [U-13C] glucose (Sigma-Aldrich, 389374), the medium was replaced with supplemented MEM with 2 g l^−1^ glucose total of which 50% was [U-13C] glucose. Approximately 2.0 × 10^9^ cells were harvested by incubation with trypsin for 5 min at 37 °C (Gibco, 25200-056) and adjusted for total protein amount (Thermo Scientific Pierce, 23227). Intracellular polar metabolites were extracted by rapid quenching with 50% methanol at −40 °C; total lipids were extracted using chloroform (Sigma-Aldrich, 366919). The cell extracts were dried by vacuum evaporation. Organic acids were derivatized to form the corresponding oximes and trimethylsilyl derivatives. Acyl-carnitines were derivatized to their corresponding methyl esters. Polar metabolites were dissolved in 99.9% 2H_2_O with 0.75% 3-(trimethylsilyl)propionic-2,2,3,3-d4 acid (Sigma-Aldrich, 293040). Lipophilic metabolites were dissolved in 2H-chloroform with 1% tetramethylsilane (Sigma-Aldrich, 151831).

### Drug treatment

Cells were exposed for a 24 h period to varying drug concentrations (as indicated in their respective citations below) while control cells were exposed to the corresponding DMSO (CAS 67-68-5, Sigma-Aldrich, D8418) dilution: metformin (glucophage; CAS 1115-70-4, 15169101, MP Biomedicals, stock 3 M in PBS), genistein (CAS 446-72-0, 10005167100 Cayman Chemicals, stock 100 mM in DMSO), haloperidol (haldol; CAS 52-86-8, 15369690 MP Biomedicals, stock 50 mM in DMSO). For acyl-carnitine measurements, the medium was supplemented with 1 mM L-carnitine (CAS 6645-46-1, Sigma-Aldrich, C0283). Each experiment was performed in three biological repeats.

### NMR spectroscopy

NMR experiments were performed on a 500-MHz Bruker Avance spectrometer with a 5-mm TXI z-gradient probe (Bruker-BioSpin, Karlsruhe, Germany) at 298 K. 13C enrichments were determined by one-dimensional 1H NMR difference spectroscopy (13C-coupled spectrum minus the 13C-decoupled spectrum). Two-dimensional indirect-detected 13C, 1H J-resolved HSQC spectra were recorded over an experimental time of 1.5 d per spectrum with 5,120 indirect 13C data points, at 80 p.p.m. 13C sweep width, 40 p.p.m. carrier position, 4,096 direct 1H increments, 3 s recycling delay and 8 scans. Before Fourier transformation, the data were multiplied with a squared sine-bell window function, phase corrected and zero-filled to 8,192 data points, for indirect 13C sampling.

### Mass spectrometry

Quantification of organic acids was accomplished by gas chromatography-mass spectrometry (ThermoFisher Trace GC Ultra/DSQ II single quadrupole mass spectrometer). Calibration standards were prepared in water and spiked with stable isotope-labelled internal standards. Detecting the derivatized organic acids was achieved by single ion monitoring of each derivative after gas chromatography separation. Acyl-carnitines were quantificated by LC/MS/MS (Waters Ultra Performance LC/triple quadrupole mass spectrometer) using flow injection analysis with electrospray ionization. Calibration standards of the acyl-carnitines were prepared in bovine serum spiked with stable isotope-labelled internal standards. Parent ion scanning was used to detect parent, acyl-carnitine molecular ions that produced a characteristic acyl-carnitine fragment ion, *m*/*z*=99, formed by collision-induced dissociation.

### Enzymatic assays

Pyridoxal 5′-phosphate or vitamin B6 was determined in an enzymatic plasma assay (A/C Diagnostics, ACB6001), which we adapted to quantify tissue culture supernatant or intracellular Vitamin B6 content by using PBST buffer with 0.1% Tween-20 (CAS 9005-64-5, Sigma-Aldrich, P9416).

### Additional methods for analysis of metformin response

Acyl-carnitine species from 2 to 16 carbons and organic acids of the tricarboxylic acid cycle were analysed by liquid chromatography mass spectrometry and show dose-dependent associations upon metformin treatment in MCF-7 cells. Acetyl-carnitine (C2) values were determined in nmol mg^−1^ protein (scaled by factor 1/1,000) and were compared with acyl-carnitine (C12-C16) levels in pmol mg^−1^ protein. Organic acids were determined in nmol mg^−1^ protein.

## Additional information

**How to cite this article:** Zielinski, D. C. *et al*. Pharmacogenomic and clinical data link non-pharmacokinetic metabolic dysregulation to drug side effect pathogenesis. *Nat. Commun.* 6:7101 doi: 10.1038/ncomms8101 (2015).

## Supplementary Material

Supplementary Information Supplementary Table 1, Supplementary Notes 1-4 and Supplementary References

Supplementary Data 1 Results of a number of computational analyses conducted in the manuscript, as well as experimental data. First worksheet of the file is a table of contents

Supplementary Data 2 Tables of the Metabolism Disease Database (MDDB) used for validation of computational predictions in the manuscript. First worksheet of the file is a table of contents.

## Figures and Tables

**Table 1:** Summary of large-scale validation of drug-metabolic perturbation associations predicted by MetChange calculations.

Property	Database	# Drugs	Properties analysed	Statistical significance
**Drug target distance**	DrugBank	134	536 Target reactions	*P*=1.65 × 10^−10^(Wilcoxon rank sum test)
**Drug–protein association**	PubMed	645	501 Signalling proteins	*P*<0.01 (Permutation test)
**Drug–metabolite association**	PubMed	645	375 Metabolites	*P*<0.001 (Permutation test)
**Drug side effect**	SIDER, PubMed	334	357 Side effects total299 For co-term analysis	*P*=5.6 × 10^−2^ (Permutation test)

Abbreviation: SIDER, Side Effect Resource.

**Table 2:** Existing studies of pharmacodynamic-altering side effect susceptibility genes and overlapping DISLoDGED pathways*.

1Side effect	2*DISLoDGED* Pathway (# metabolites in pathway)	3PD gene	4Nutrient overlap	5Patho-physiology	6Metabolic perturbation	7*In vivo* drug perturbation	8Supplement
Weight gain	Melatonin	*MC4R*	*MC4R* levels correlated with melatonin	Melatonin and *MC4R* energy regulators	Decreased melatonin tied to metabolic syndrome	Antipsychotics suppress melatonin synthesis	Melatonin
Parkinsonism	Poly-unsaturated fatty acids	*ZNF202*	*ZNF202* lipid regulator	PUFAs linked to Lewy bodies	Altered lipid oxidation	RBC PUFAs altered	Essential fatty acids
Tardive Dyskinesia	Serotonin	5-HT receptors	Direct ligand	Dopaminergic neuron interaction	NA	5-Hydroxy-indoleacetate perturbation	Conflicting findings
Tardive Dyskinesia	Ascorbate	D3 receptors	Dopamine synthesis cofactor	Dopamine primary mediator	NA	Metabolite levels perturbed	Vitamin C, vitamin E
Myotoxicity	CoQ10	*COQ2*	CoQ10 synthesis	Mitochondrial function	Q10 deficiency associated w/ myopathy	Decreased under statin treatment	CoQ10 (conflicting)
Cardiac arrhythmia	Urea cycle	*NOS1AP*	Nitrogen regulation	NO affects cardiac function	Altered NO linked to arrhythmia	Various affect NO production	L-arginine
Cardiac arrhythmia	Pentose phosphate pathway	*GPD1L*, *ZFHX3*	Oxidative stress response	Oxidative stress affects cardiac function	H_2_O_2_ induces arrhythmia	Clomipramine induces via oxidative stress	Various antioxidants
Hearing loss	Ascorbate	*COMT*	Dopamine synthesis cofactor	Dopamine innervation in inner ear	Dopa synth. inhibition tied to hearing loss	Cisplatin inhibits dopamine	Vitamin C, dopamine
Hearing loss	Ascorbate, lipoate	Glutathione S-transferases	Oxidative stress response	ROS-induced cochlear cell death	SOD deficiency tied to hearing loss	Cisplatin induces via oxidative stress	Vitamin C, lipoate

Abbreviations: NA, not applicable; PUFA, poly-unsaturated fatty acids; RBC, red blood cell; ROS, reactive oxygen species; SOD, superoxide dismutase; 5-HT, serotonin.

^*^Existing studies of pharmacodynamic-altering side effect susceptibility genes and overlapping DISLoDGED pathways. The two cases of tardive dyskinesia have ‘NA' in the ‘Metabolic perturbation in disease' row as tardive dyskinesia is not typically described as a disease outside of its occurrence as an adverse drug response, therefore there is no drug-independent case for comparison. See the Supplementary Note 1 for full discussion and references related to each case.
