# Supplementary material for: Pharmacogenomic and clinical data link non-pharmacokinetic metabolic dysregulation to drug side effect pathogenesis
Source: Nat Commun. 2015 Jun 9;6:7101. doi: 10.1038/ncomms8101 (PMC4468904; doi:10.1038/ncomms8101)
Supplement: Supplementary Information — Supplementary Table 1, Supplementary Notes 1-4 and Supplementary References [file ncomms8101-s1.pdf]

	Subsystem	Subset	Coverage	p-value
ADR-associated gene transcription perturbations	Vitamin metabolism	Both	enriched	2.0×10^{-5} / 2.4×10^{-2}
	Amino acid metabolism	Both	enriched	5.2×10^{-3} / 2.5×10^{-4}
	Glycosylation metabolism	Both	depleted	3.7×10^{-4} / 6.9×10^{-3}
	Nucleotide metabolism	Both	depleted	3.9×10^{-2} / 4.5×10^{-2}
	Unsaturated fatty acid metabolism	Down	enriched	2.4×10^{-3} / -
	Inositol metabolism	Down	enriched	1.8×10^{-2} / -
	Fatty acid metabolism	Down	depleted	4.7×10^{-3} / -
	Lipid metabolism	Down	depleted	3.3×10^{-2} / -
	ROS metabolism	Up	enriched	- / 1.9×10^{-2}
	Glycogen metabolism	Up	enriched	- / 3.8×10^{-2}
Disease-associated metabolic genes	Transport	N/A	enriched	4.7×10^{-3}
	Inositol metabolism	N/A	enriched	2.0×10^{-2}
	Central metabolism	N/A	depleted	3.5×10^{-2}

Supplementary Table 1: Summary of pathway enrichment results. This table contains a summary of the pathway enrichment results of both side effect-associated gene expression perturbations from MetChange analysis of the Connectivity Map database (above) and disease-linked genes from GWAS studies (below). The two sets show similar enrichment patterns, with a general lack of enrichment in central carbon metabolism and notably enrichment of inositol metabolism in side effect-linked down-regulations and disease-linked genes. p-values shown are one-tailed hypergeometric tests.

Supplementary Note 1

Antipsychotic-induced weight gain

a. *Drugs with side effect (corresponding frequencies):*
   i. *Weight increase:* Citalopram (0.505), clozapine (0.36556), letrozole (0.051578), testosterone (0.0005), nilutamide (0.0005), levonorgestrel (0.0005), bupropion (0.0005)
   ii. *Weight gain:* Fluvoxamine (0.505), fluoxetine (0.41973), paroxetine (0.20188), mitoxantrone (0.14017), pergolide (0.053125), clozapine (0.04), riluzole (0.20188) buspirone (0.0055), terazosin (0.000533), vigabatrin (0.0005), ramipril (0.0005), omeprazole (0.0005), nabumetone (0.0005), lansoprazole (0.0005), ketoprofen (0.0005), fenofibrate (0.0005), cyclobenzaprine (0.0005), bisoprolol (0.0005)
b. *Observed Perturbations (direction) (overlaps with gene in* ***bold****):*
   i. *Weight increase:* 1 acyl phosphoglycerol (up), **melatonin (down)**, propionyl-carnitine (up), thiosulfate (up)
   ii. *Weight gain*: 12R-Hydroperoxyeicosatetraenoate (down), choline phosphate (down), propionyl-carnitine (up), sulfate (up)
c. *Gene:* Variants in *MC4R*, which codes a G protein-coupled receptor of the melanocortin family that binds to the peptide alpha melanocyte-stimulating hormone (AMSH), have been shown to be associated with weight gain induced by second-generation antipsychotics[1]
   i. *Relevant perturbation:* melatonin (down)
   ii. *Overlaps with gene pathway:* Due to the primary role of MC4R as a receptor for the peptide AMSH there is no direct pathway to which to assign MC4R. However, melatonin levels have been shown in vertebrates to correlate with levels of both MC4R and leptin, the latter of which independently increases expression of AMSH, suggesting direct functional overlap between melatonin levels and MC4R activity[2]. We did not find any other modulators of MC4R reported in the literature, though they may exist, and thus considered melatonin as the primary metabolic pathway overlapping with MC4R. Metabolites involved in melatonin synthesis and degradation starting with tryptophan compose a set of 6/344 metabolites.
   iii. *Relevance of the perturbation to the physiology of the side effect pathology:* As melatonin regulates activity of MC4R and leptin, both established energy and appetite regulators[3, 4], possible physiological connections between melatonin deficiency and anti-psychotic weight gain are clear.
   iv. *Perturbation associated with the pathology:* Independently of studies targeting its effect on MC4R, decreased melatonin has been associated with sleep deprivation-associated obesity and metabolic syndrome[5], further suggesting a direct link between melatonin deficiency and the pathology of antipsychotic-induced weight gain.
   v. *Known supplements targeted at perturbed pathways:* Melatonin replacement therapy has been shown to block weight gain induced by the antipsychotic drug olanzapine in rats[6], further suggesting that deficiency may be causal of the pathology.
   vi. In vivo *occurrence of metabolic perturbation:* The antipsychotic drugs chlorpromazine and haloperidol have been shown to suppress activity of acetylserotonin O-methyltransferase, the ultimate step in melatonin synthesis, in bovine and sheep pineal

glands, and thus the cell culture side effect-linked drug response is recapitulated *in vivo* in this case[7].

Antipsychotic-induced Parkinsonism

a. *Drugs with side effect (corresponding frequencies):* Memantine (0.0055), selegiline (0.0005), buspirone (0.0005), valproic acid (0.00045)
b. *Observed Perturbations (direction) (overlaps with gene in **bold**):* 1D-myo-Inositol 3,4-bisphosphate (up), **choline phosphate (up),** D-xylose (down), L-homocysteine (up), melatonin (down), **prostaglandin E1 (down)**, sodium (down)
c. *Gene:* Variants of *ZNF202*, which codes a broad lipid regulating transcription factor[8] with targets including the phospholipid transporter ABCA1[9], have been associated with antipsychotic-induced movement disorders including Parkinsonism[10]. Additionally, another study found genes in lipid-associated pathways, among others, to be significantly associated with antipsychotic-induced extrapyramidal symptoms, but these SNPs failed to reach a GWAS significance level[11].
    i. *Relevant perturbation:* choline phosphate (up), prostaglandin E1 (down)
    ii. *Overlaps with gene pathway:* We observed two Parkinsonism-linked metabolic gene expression perturbations in lipid pathways, including the phospholipid precursor choline phosphate and a poly-unsaturated fatty acid (PUFA), PGE1. As ZNF202 is known the regulator broad classes of lipids, we included all lipid pathways as potential overlapping pathways, a set of 73/344 metabolites.
    iii. *Relevance of the perturbation to the physiology of the side effect pathology:* Multiple physiological ties between altered lipid metabolism and the pathology of Parkinson's disease have been suggested, most notably the link between the lipid binding protein alpha-synuclein, a key component of Lewy bodies[12], and both altered phospholipids[13] and altered PUFAs [14]. Additionally, the disruption of mitochondrial function associated with perturbation of PUFAs has been proposed to play a role[14].
    iv. *Perturbation associated with the pathology:* Oxidative stress and oxidized lipid products been associated with Parkinson's disease[15]. Substantial lipid alterations are known to occur in the brain during onset of Parkinson's disease[16]. Dietary unsaturated fatty acid intake has been shown to affect risk for Parkinson's disease[17].
    v. *Known supplements targeted at perturbed pathways:* Multiple studies have shown essential fatty acids to be a beneficial supplement in the treatment of Parkinson's disease[18, 19].
    vi. In vivo *occurrence of metabolic perturbation:* Essential fatty acid levels have been shown to be altered in red blood cells *in vivo* under antipsychotic treatment[20], showing that the Parkinsonism-linked perturbation seen in cell culture has been shown to occur *in vivo* as well.

Antipsychotic-induced tardive dyskinesia

a. *Drugs with side effect (corresponding frequencies):* Risperidone (0.00091237), valproic acid (0.0005), pimozide (0.0005), fluvoxamine (0.0005), clozapine (0.0005), citalopram (0.0005), bupropion (0.0005)
b. *Observed Perturbations (direction) (overlaps with gene in **bold**):* 2,4 dihydroxy nitrophenol (down), ethanolamine phosphate (up), Fe2+ (up), **formyl-N-acetyl-5-methoxykynurenamine (down),** lipoate (down), O-phospho-L-serine (up), **oxalate (up),** sodium (down)
c. Gene: Variants of serotonin (5-HT) receptors, including 5-HT2A[21], have been associated with susceptibility to tardive dyskinesia as an adverse effect of antipsychotic treatment
    i. *Relevant perturbation:* formyl-N-acetyl-5-methoxykynurenamine (down)
    ii. *Overlaps with gene pathway:* We observe a down-regulation of formyl-N-acetyl-5-methoxykynurenamine, the product of a five reaction biosynthetic pathway from tryptophan that includes serotonin. From this pathway, a set of 6/344 metabolites is obtained, as with the MC4R case above.
    iii. *Relevance of the perturbation to the physiology of the side effect pathology:* While dopaminergic neuron activity thought to play a central role in the development of tardive dyskinesia, the pathogenesis is not fully elucidated. Serotonin receptors are thought to interact with dopaminergic neurons in tardive dyskinesia[22], and down-regulation of 5-HT production has been found to be sufficient, though not necessary, for the alleviation of tardive dyskinesia that occurs with deep brain stimulation therapy[23].
    iv. *Perturbation associated with the pathology:* Tardive dyskinesia is not normally described as a pathology independent of drug treatment. That said, serotonin is thought to play a role in tardive dyskinesia[22], and agonists of the serotonin receptor 5-HT1A have been shown to alleviate tardive dyskinesia, suggesting the serotonin pathway is involved in the pathology or at least affects it[24].
    v. *Known supplements targeted at perturbed pathways:* Results are conflicting as to whether increased or decreased serotonin activity is beneficial, as mentioned above, with decreased 5-HT activity[23], 5-HT3 antagonism[25] and 5-HT1A agonism[24] all being associated with effective treatment of tardive dyskinesia. A small five patient study testing the efficacy of the serotonin precursor 5-hydroxytryptophan showed no improvement in tardive dyskinesia[26], which may have been expected given several studies showing decreased 5-HT activity associated with alleviated tardive dyskinesia.
    vi. In vivo *occurrence of metabolic perturbation:* Levels of the serotonin metabolite 5-hydroxyindoleacetate has been shown to be perturbed in brain tissues of rats with tardive dyskinesia induced by treatment with the antipsychotic haloperidol[27].

a. Gene: Variants of the D3 dopamine receptor have been shown to be associated with tardive dyskinesia[28]. One other study did find no association between certain dopamine receptor variants and tardive dyskinesia occurrence[29].
    i. *Relevant perturbation:* oxalate (up)
    ii. *Overlaps with gene pathway:* Dopamine is synthesized in a sequential set of reactions downstream of tyrosine and converted to norepinephrine by dopamine beta-monooxygenase, an L-ascorbate (vitamin C)-dependent enzyme. Including the entire

subnetwork as well as the cofactors vitamin C, tetrahydrobiopterin, and S-adenosyl-L-methionine metabolic subnetworks, we obtain a set of 16/344 overlapping metabolites.

iii. *Relevance of the perturbation to the physiology of the side effect pathology:* Dopamine receptors are thought to play a primary role in the pathogenesis of antipsychotic-induced tardive dyskinesia, as many antipsychotics are dopamine antagonists. We observe an up-regulation of the vitamin C metabolite oxalate, indicating an up-regulation of vitamin C metabolism and therefore a possible depletion of L-ascorbate. As L-ascorbate is a cofactor required in the metabolism of dopamine, it is possible that perturbations in vitamin C processing could impact dopamine metabolism. Supporting this, multiple studies have shown that vitamin C has an antidopaminergic effect in combination with antipsychotics[30, 31], consistent with greater L-ascorbate enhancing dopamine degradation. Thus, it is possible that antipsychotic-induced depletion of vitamin C may alter the drug effect on dopamine receptors, leading to the side effect.

iv. *Perturbation associated with the pathology:* We did not find any links between vitamin C deficiency and tardive dyskinesia independent of drug treatment, as the pathology of the side effect is not reported independently of drug treatment.

v. *Known supplements targeted at perturbed pathways:* Vitamin C supplementation has been shown in combination with Vitamin E to be an effective supplement for tardive dyskinesia, although this effect is thought to be primarily due to antioxidative effects of the vitamins[32]. Furthermore, vitamin C supplementation has been found to improve outcome and decrease stress associated with atypical antipsychotics[33].

vii. In vivo *occurrence of metabolic perturbation:* Vitamin C has been found to be diminished in schizophrenics, and it is proposed the schizophrenics require greater vitamin C intake than healthy patients[34, 35]. This is consistent with the up-regulated vitamin C degradation pathway seen in drugs that induce tardive dyskinesia, a list dominated by antipsychotics. Furthermore, levels of dopamine metabolites have been shown to be perturbed in brain tissues of rats with tardive dyskinesia induced by treatment with the antipsychotic haloperidol[27].

Drug-induced arrhythmia

a. *Drugs with side effect (corresponding frequencies):* Theophylline (0.1525), aminophylline (0.13667), quinidine (0.03) mitoxantrone (0.024118), galantamine (0.0055), clomipramine (0.0055), fluoxetine (0.0029706), citalopram (0.0027807) doxazosin (0.0017143), sulindac (0.0005), ramipril (0.0005), paroxetine (0.0005), paclitaxel (0.0005), oxybutynin (0.0005), naproxen (0.0005), nabumetone (0.0005), levonorgestrel (0.0005), lansoprazole (0.0005), ketoprofen (0.0005), iodixanol (0.0005), indapamide (0.0005), fluvoxamine (0.0005), flurbiprofen (0.0005), fenofibrate (0.0005), felodipine (0.0005), etodolac (0.0005), diltiazem (0.0005), diclofenac (0.0005), cyclobenzaprine (0.0005), ciprofloxacin (0.0005), cefotaxime (0.0005), amoxicillin (0.0005), amoxapine (0.0005), amantadine (0.0005), salbutamol (0.00036364), propofol (0.00033333), carbamazepine (0.0003), norfloxacin (0.00015)

b. *Observed Perturbations (direction) (overlaps with gene in* ***bold****):* **D-Ribulose 5-phosphate (down)**, **L-glutamate 5-phosphate (up)**, prostaglandin E1 (up), UDP-D-xylose (down), **urea (up)**

c. *Gene:* Rare variants in both Glycerol-3-phosphate dehydrogenase 1-like protein (*GPD1L*) and Zinc finger homeobox 3 (*ZFHX3*) have been associated with drug-induced torsades de pointes[36]. Additionally, *GPD1L* mutations cause Brugada syndrome type 2, a disorder characterized by cardiac arrhythmia[37], and *ZFHX3* mutation has been associated with atrial fibrillation[38]. We group these genes together due to their overlapping role in regulation of oxidative stress response, as described below.

   i. *Relevant perturbation:* D-Ribulose 5-phosphate (down)

   ii. *Overlaps with gene pathway:* GLPD1 has been shown to be a metabolic oxidative state sensor in the heart[39]. Similarly, ZFHX3 is an oxidative stress response regulator[40] whose mutation is associated with cardiac arrhythmia. Ribulose-5-phosphate is the end product of the oxidative branch of the pentose phosphate pathway that is responsible for a large fraction of NADPH production and maintenance of the reduced glutathione pool and hence the oxidative state of the cell. Thus, we observe down-regulated oxidative pentose phosphate pathway in drugs causing arrhythmia, which overlaps with the known susceptibility genes with functions in sensing cellular oxidation state. We attempted to include all pathways related to oxidative stress, including pentose phosphate pathway, the primary generator of glutathione, hydrogen peroxide, and antioxidant pathways including vitamin C, glutathione, melatonin, lipoate, and coenzyme Q10, a set of 30/344 metabolites.

   iii. *Relevance of the perturbation to the physiology of the side effect pathology:* Oxidative state, through NADH state, has been shown to influence the PKC-dependent phosphorylation of the sodium channel SCN5A in GPD1L-linked arrhythmias[39]. Similarly, oxidative stress has been shown to alter K+ currents in the heart[41] in a glutathione-dependent manner. Additionally, oxidative stress is thought to affect mitochondrial energetics in the heart, contributing to arrhythmias[42-44].

   iv. *Perturbation associated with the pathology:* Hydrogen peroxide has been shown to induced cardiac arrhythmias in guinea pig hearts[45], suggesting oxidative stress can induce arrhythmias directly.

   v. *Known supplements targeted at perturbed pathways:*
   Vitamin A and Vitamin C both have been shown to reduce epinephrine-induced arrhythmias[46]. Several antioxidants have been shown effective in reducing reperfusion-induced arrhythmias, including the glutathione precursor N-acetylcysteine [47], taurine[48, 49], and melatonin[50]. Additionally, the antioxidant coenzyme Q10 has been shown to improve outcome following congestive heart failure[51].

   vi. In vivo *occurrence of metabolic perturbation:* Clomipramine, an antidepressant with one of the highest reported incidences of induced arrhythmia according to the SIDER database, has been shown to induce cardiotoxicity in an oxidative stress-dependent manner[52], and thus the arrhythmia-linked perturbation in cell culture appears relevant to *in vivo* drug response in at least one case.

d. *Gene:* Variants of *NOS1AP*, encoding nitric oxide synthase 1 adaptor protein, are associated with drug-induced long QT syndrome and ventricular arrhythmia[53]
    i. *Relevant perturbation:* L-glutamate 5-phosphate (up), urea (up)
    ii. *Overlaps with gene pathway:* NOS1AP binds neuronal nitric oxide synthase[54], a key regulator of NO signaling in the heart[55]. We observe two up-regulations around the urea cycle and arginine metabolism (L-glutamate 5-phosphate and urea), which connects nitric oxide, glutamate, creatine, urea, and polyamine metabolism. As NOS1AP is a nitrogen regulator, we included metabolites involved in the urea cycle, including creatine and nitric oxide, downstream of glutamate and until the polyamine precursor L-ornithine and the first polyamine putrescine, a set of 12/344 metabolites.
    iii. *Relevance of the perturbation to the physiology of the side effect pathology:* Neuronical nitric oxide synthase (nNOS), which is bound by NOS1AP, regulates cardiac function in various ways[55]. NO is reported to affect cardiac ion channels and signaling pathways in a variety of ways[56].
    iv. *Perturbation associated with the pathology:* Decreases in cardiac function in cirrhosis have been shown to be NO dependent[57]. Inversely, cirrhotic rats have been found to have increased resistance to epinephrine-induced arrhythmia due to constitutively higher NO production[58].
    v. *Known supplements targeted at perturbed pathways:* Supplementation with L-arginine has been shown to diminish arrhythmia scores in digoxin-treated isolated heart experiments[59].
    i. In vivo *occurrence of metabolic perturbation:* Theophylline, the drug with the highest reported incidence of arrhythmia, has been shown to inhibit arginine-dependent production of NO[60]. Similarly, serotonin blockers such as clomipramine (which, as mentioned above, is associated with a high incidence of arrhythmia) have been shown to inhibit nitric oxide synthase[61]. These findings are consistent with altered nitrogen metabolism observed in cell culture response.

e. *Links between pathology and other observed metabolic perturbations:* As an interesting note, in addition to pentose phosphate pathway and nitrogen metabolism, we also observed linked between the other observed arrhythmia-linked metabolic perturbations in eicosanoid metabolism and glycolysation and the pathogenesis of cardiac arrhythmias. For example, we observe a down-regulation of the glycosylation precursor UDP-D-xylose, and altered ion channel glycosylation has been shown to be one mechanism important to drug-induced arrhythmia[62]. Furthermore, we observe an up-regulation of prostaglandin E_1, which has been shown to be a therapeutic supplement to arrhythmias[63, 64]. Additionally, prostaglandins have been shown to affect cardiac electrophysiology[65], and disturbed prostaglandin ratios post-infarction are proposed to play a role in occurrence of arrhythmias[66].

Statin-induced myotoxicity

a. *Drugs with side effect (corresponding frequencies):*
    i. *Asthenia:* Galantamine (0.505), irinotecan (0.44), etoposide (0.39), gabapentin (0.28852), ribavirin (0.27292), atovaquone (0.1875), fluvoxamine (0.18671), riluzole (0.16575), doxorubicin (0.14406), mitoxantrone (0.12), thalidomide (0.09675), carbamazepine

(0.076667), pergolide (0.060875), letrozole (0.044204), valproic acid (0.04025), flecainide (0.03375), quinidine (0.02), salbutamol (0.017182), testosterone (0.013333), baclofen (0.0108), lisinopril (0.01), enalapril (0.009), timolol (0.0071667), zidovudine (0.0048889), fenofibrate (0.0029716), simvastatin (0.0021821), paclitaxel (0.0005), oxybutynin (0.0005), nifedipine (0.0005), naproxen (0.0005), naltrexone (0.0005), nabumetone (0.0005), mefloquine (0.0005), lidocaine (0.0005), lansoprazole (0.0005), fulvestrant (0.0005), fluvastatin (0.0005), flurbiprofen (0.0005), finasteride (0.0005), diclofenac (0.0005), cyclobenzaprine (0.0005), ciprofloxacin (0.0005), propofol (0.00033333)

ii. *Muscle weakness:* Clozapine (0.01), risperidone (0.0055), dihydroergotamine (0.0055), citalopram (0.0055), propafenone (0.0005), omeprazole (0.0005), naproxen (0.0005), mefloquine (0.0005), fluvastatin (0.0005), bupropion (0.0005), amiodarone (0.0005), doxazosin (0.00044444), simvastatin (0.00016667)

iii. *Myalgia:* Ribavirin (0.56929), selegiline (0.505), progesterone (0.08), daunorubicin (0.063333), pergolide (0.05875), mitoxantrone (0.037608), letrozole (0.035423), salbutamol (0.017455), verapamil (0.011), dihydroergotamine (0.0055), paclitaxel (0.0039286), doxazosin (0.0018333), testosterone (0.0005), terazosin (0.0005), ranitidine (0.0005), ramipril (0.0005), procarbazine (0.0005), omeprazole (0.0005), ofloxacin (0.0005), naproxen (0.0005), naltrexone (0.0005), mefloquine (0.0005), leflunomide (0.0005), lansoprazole (0.0005), ketorolac (0.0005), ketoprofen (0.0005), guanfacine (0.0005), fluoxetine (0.0005), fenofibrate (0.0005), felodipine (0.0005), dipyridamole (0.0005), diclofenac (0.0005), cyclobenzaprine (0.0005), ciprofloxacin (0.0005), cetirizine (0.0005), propofol (0.00033333), norfloxacin (0.000125)

iv. *Myopathy:* Paroxetine (0.0005), lovastatin (0.0005), fluvoxamine (0.0005), fluvastatin (0.0005), fluoxetine (0.0005), diltiazem (0.0005), clomipramine (0.0005), amiodarone (0.0005)

v. *Myositis:* Pergolide (0.0055), paroxetine (0.0005), ofloxacin (0.0005), lansoprazole (0.0005), fluvastatin (0.0005), fluoxetine (0.0005), fenofibrate (0.0005), clomipramine (0.0005)

vi. *Rhabdomyolysis:* Aminophylline (0.046667), theophylline (0.035), ofloxacin (0.0005), lovastatin (0.0005), indapamide (0.0005), ganciclovir (0.0005), fluvastatin (0.0005), fenofibrate (0.0005), clozapine (0.0005), citalopram (0.0005), ciprofloxacin (0.0005), bupropion (0.0005), amiodarone (0.0005), propofol (0.00025), simvastatin (0.00017742)

b. *Observed Perturbations (direction) (overlaps with gene in* ***bold****):* Several side effects related to myotoxicity were analyzed.

i. *Asthenia:* 1D-myo-Inositol 1,4-bisphosphate (up), biocytin (down), D-glucose 6-phosphate (up), D-xylose (down), flavin adenine dinucleotide oxidized (down), glycogen, structure 3 (glycogenin-7[1,4-Glc]) (up), homogentisate (up), L-homocysteine (up), oxaloacetate (down), phosphocreatine (up), prostaglandin E2 (up), prostaglandin F2alpha (down)

ii. *Muscle weakness:* 1 acyl phosphoglycerol (up), 1D-myo-Inositol 4-phosphate (down), diacylglycerol (homo sapiens) (down), hyaluronan biosynthesis, precursor 1 (down)

iii. *Myalgia:* 2-methyl-3-oxopropanoate (down), dolichol phosphate, human uterine homolog (up), peptide sans lysine (down), serotonin (up)
iv. *Myopathy:* 24R,25-Dihyoxyvitamin D2 (down), epiandrosterone (up), N-acetyl-D-glucosamine (up)
v. *Myositis:* D-lactaldehyde (up), sulfate (up), **ubiquinol-10 (down)**
vi. *Rhabdomyolysis:* Ammonium (up), D-mannose (up), hydroxy coumarin (down), phylloquinone (down)

c. *Gene: COQ2*, encoding parahydroxybenzoate--polyprenyl transferase, a gene in ubiquinone synthesis, has been associated with various statin myopathies[67], including myositis
   i. *Relevant perturbation:* ubiquinol-10 (down)
   ii. *Overlaps with gene pathway:* Metabolites involved in the coenzyme Q10 synthesis pathway and forms of coenzyme Q10 are considered to be associated with COQ2, however, from this pathway only ubiquinol-10 itself appears as a potential prediction of the method, resulting in only 1/344 metabolites overlapping with the susceptibility gene.
   iii. *Relevance of the perturbation to the physiology of the side effect pathology:* Mitochondrial dysfunction is thought to play a role in statin-induced myopathies, and as coenzyme Q10 plays a key role in proper mitochondrial function, induction of Q10 deficiency could clearly play a causal role in disrupting mitochondrial function[68, 69].
   iv. *Perturbation associated with the pathology:* Certain coenzyme Q10 deficiencies are known to be associated with myopathies[70]. Mitochondrial myopathy has been found to be associated with coenzyme Q10 deficiency as well[71], and Q10 supplementation has been found to alleviate this form of myopathy[72].
   v. *Known supplements targeted at perturbed pathways:* Interestingly, the use of Q10 has been shown effective in relieving statin-induced myopathies in some studies[73, 74] and ineffective in others[75]. We note that specifically Q10 has been shown to alleviate muscle inflammation (myositis) following exercise independent of statin treatment, suggesting the possibility that the beneficial effects of Q10 are mediated through anti-inflammatory effects[76], while other pathological factors may be in play in other myopathies.
   vi. In vivo *occurrence of metabolic perturbation:* Levels of coenzyme Q10 have been shown in various studies to be decreased in patients undergoing statin treatment[77-81].

Cisplatin-induced hearing loss

a. *Drugs with side effect (corresponding frequencies):* Pergolide (0.0055), memantine (0.0055), gabapentin (0.0055), clomipramine (0.0055), risperidone (0.0049444), fluvoxamine (0.0012143), tobramycin (0.0005), riluzole (0.0005), ribavirin (0.0005), ramipril (0.0005), procarbazine (0.0005), paroxetine (0.0005), naproxen (0.0005), mefloquine (0.0005), lansoprazole (0.0005), ketorolac (0.0005), ketoprofen (0.0005), flurbiprofen (0.0005), fluoxetine (0.0005), etodolac (0.0005), diflunisal (0.0005), diclofenac (0.0005), ciprofloxacin (0.0005), bupropion (0.0005), bisoprolol (0.0005), amoxicillin (0.0005), valproic acid (0.00040909), norfloxacin (0.000125)
b. *Observed Perturbations (direction) (overlaps with gene in* ***bold****):* 2,4 dihydroxy nitrophenol (down), **lipoate (down)**, thiamin monophosphate (down), **L-threonate (down)**
c. Gene: Variants of catechol O-methyltransferase (COMT) have been associated with cisplatin-induced hearing loss[82].

i. *Relevant perturbation:* L-threonate (down)

ii. *Overlaps with gene pathway:* L-threonate is a metabolite of L-ascorbate. COMT is involved in the metabolism of catecholamines, including dopamine, epinephrine, and norepinephrine. Dopamine is synthesized in a sequential set of reactions downstream of tyrosine and converted to norepinephrine by dopamine beta-monooxygenase, an L-ascorbate (vitamin C)-dependent enzyme. Including the entire subnetwork as well as the cofactors vitamin C, tetrahydrobiopterin, and S-adenosyl-L-methionine metabolic subnetworks, we obtain a set of 16/344 overlapping metabolites. The vitamin C pathway, where we observed a perturbation, thus overlaps with COMT through its role as a cofactor for dopamine beta hydroxylase.

iii. *Relevance of the perturbation to the physiology of the side effect pathology:* There is significant dopaminergic neuron innervation in the inner ear[83], and thus proper dopamine metabolism is essential for function. D2/D3 receptors have been found to play an important role in hearing, with dopamine agonists playing a protective role in hearing loss[84]. The production of norepinephrine from dopamine is catalyzed by dopamine beta hydroxylase, which requires vitamin C as a substrate.

iv. *Perturbation associated with the pathology:* Inhibition of vitamin C transport has been reported to cause inhibition of catecholamine production[85], which interferes with proper dopaminergic transmission. Additionally, mice deficient in dopamine beta hydroxylase, an enzyme in dopamine metabolism that uses vitamin C as a cofactor, have been reported to be subject to acquired hearing loss.

v. *Known supplements targeted at perturbed pathways:* Vitamin C has been shown to be an effective supplement in preventing damage-induced hearing loss[86]. Vitamin C has even specifically been shown to inhibit cisplatin toxicity[87]. In addition, dopamine has been shown to have a protective effect in hearing loss and is thought to be a potentially effective therapeutic supplement[88].

vi. In vivo *occurrence of metabolic perturbation:* Cisplatin has been shown *in vivo* in mammals to inhibit secretion of catecholamines[89], although inhibition of vitamin C metabolism as a possible mechanism for this has not been directly investigated.

d. Gene: Variants of glutathione-S-transferases[90] have been shown to be associated with cisplatin-induced hearing loss.

i. *Relevant perturbation:* L-threonate (down), lipoate (down)

ii. *Overlaps with gene pathway:* Glutathione-s-transferases are involved in oxidative stress response, so we attempted to include all pathways related to oxidative stress, including pentose phosphate pathway, the primary generator of glutathione, hydrogen peroxide, and antioxidant pathways including vitamin C, glutathione, melatonin, lipoate, and coenzyme Q10, a set of 30/344 metabolites. The vitamin C and lipoate pathways, where we observed perturbations, overlap with glutathione-S-transferase for their role as general antioxidants.

iii. *Relevance of the perturbation to the physiology of the side effect pathology:* Reactive oxygen species (ROS) have been associated with both apoptotic and necrotic death of cochlear cells in noise-induced hearing loss[91].

iv. *Perturbation associated with the pathology:* A deficiency in the ROS response protein superoxide dismutase has been associated with increased susceptibility to hearing loss[92].

v. *Known supplements targeted at perturbed pathways:* Vitamin C[86] and lipoate[93] have been shown to be effective in preventing noise- and drug-induced hearing loss, respectively, when supplemented.

vi. In vivo *occurrence of metabolic perturbation:* Cisplatin has been observed *in vivo* to cause a significant oxidative load[94], which is thought to be a causal factor in its ototoxicity.

Supplementary Note 2

Calculation of joint probability of predictions overlapping with side effect susceptibility genes

As a number of perturbations were observed for each side effect, and several metabolites would be considered potentially overlapping in each case (other than with coenzyme Q10, which had a single overlapping metabolite within the set of observed side effect-linked metabolites), it is difficult to determine by inspection whether the side effect-linked metabolites are significantly predictive of genetic susceptibility pathways for corresponding side effects. To attempt to address this rigorously, we took the set of distinct side effect-linked metabolites observed in any case, a set of 344 metabolites, and the set of distinct metabolites linked to each particular side effect, and considered the latter 'draws' in a hypergeometric test. As we observed overlap in 9/9 cases, the joint probability of this occurring by chance is thus the product of the probabilities in each separate case, assessed by hypergeometric tests based on the number of metabolite perturbations and potentially overlapping metabolites in each case. This assumes that the probability of 'drawing' each metabolite is the same as drawing any other, which depends on complex factors determining the underlying prediction distribution; however, we do not expect this to greatly affect the probability, given that apparent distribution of side effect-linked metabolites is relatively uniform upon inspection.

This process resulted in the following statistical tests (p = 1 – cdf + pdf/2):

1) Antipsychotic-induced weight gain with *MC4R:* 7 distinct perturbations, 1 overlapping, 6 metabolites of 344 potentially overlapping - hypergeometric p-value = 0.0610
2) Antipsychotic-induced Parkinsonism with *ZNF202*: 7 distinct perturbations, 2 overlapping, 73 metabolites of 344 potentially overlapping - hypergeometric p-value = 0.3127
3) Antipsychotic-induced tardive dyskinesia with serotonin receptors: 8 distinct perturbations, 1 overlapping, 6 metabolites of 344 potentially overlapping - hypergeometric p-value = 0.0697
4) Antipsychotic-induced tardive dyskinesia with dopamine and adrenergic receptors: 8 distinct perturbations, 1 overlapping, 16 metabolites of 344 potentially overlapping - hypergeometric p-value = 0.1839
5) Drug-induced arrhythmia and *GPD1L*/*ZFHX3:* 5 distinct perturbations, 1 overlapping, 30 metabolites of 344 potentially overlapping - hypergeometric p-value = 0.2152
6) Drug-induced arrhythmia and *NOS1AP*: 5 distinct perturbations, 2 overlapping, 12 metabolites of 344 potentially overlapping - hypergeometric p-value = 0.0054
7) Statin-induced myotoxicity and *COQ2*: 30 distinct perturbations, 1 overlapping, 1 metabolite of 344 potentially overlapping - hypergeometric p-value = 0.0436
8) Cisplatin-induced hearing loss and *COMT*: 4 distinct perturbations, 1 overlapping, 16 metabolites of 344 potentially overlapping – hypergeometric p-value = 0.0929
9) Cisplatin-induced hearing loss and Glutathione-S-transferases: 4 distinct perturbations, 2 overlapping, 30 metabolites of 344 potentially overlapping – hypergeometric p-value = 0.0209

Joint probability of 9/9 overlapping: $0.06*0.31*0.07*0.18*0.22*0.0054*0.044*0.09*0.02 = 2.2*10^{-11}$

Thus, it is unlikely that the overlap with genetic susceptibility factors is a byproduct of the number of predictions made or the number of potentially overlapping metabolites. The corroborating *in vivo*, physiological, and supplementation data in each case further supports this.

Supplementary Note 3

Studies in which pathways related to drug metabolism and transport show perturbed gene expression associated with the side effect

Statin-induced myotoxicity

a. *Drugs with side effect (corresponding frequencies):* Same as above myotoxicity example
b. *Observed Perturbations (direction) (overlaps with gene in* ***bold****):* Same as above myotoxicity example.
   i. *Asthenia:* 1D-myo-Inositol 1,4-bisphosphate (up), biocytin (down), D-glucose 6-phosphate (up), D-xylose (down), flavin adenine dinucleotide oxidized (down), glycogen, structure 3 (glycogenin-7[1,4-Glc]) (up), homogentisate (up), L-homocysteine (up), oxaloacetate (down), phosphocreatine (up), **prostaglandin E2 (up)**, **prostaglandin F2alpha (down)**
   ii. *Myalgia:* 2-methyl-3-oxopropanoate (down), dolichol phosphate, human uterine homolog (up), peptide sans lysine (down), serotonin (up)
   iii. *Myopathy:* 24R,25-Dihyoxyvitamin D2 (down), epiandrosterone (up), N-acetyl-D-glucosamine (up)
   iv. *Myositis:* D-lactaldehyde (up), sulfate (up), ubiquinol-10 (down)
   v. *Rhabdomyolysis:* Ammonium (up), D-mannose (up), hydroxy coumarin (down), phylloquinone (down)

a. Gene: SLCO1B1, which transports several substrates including eicosanoids and statins, has been shown to be a susceptibility gene for myotoxicity[95].
   i. Relevant perturbation: prostaglandin E2 (up), prostaglandin F2alpha (down)
   ii. Overlaps with gene pathway: While SLCO1B1 has been shown to have a role in statin transport, other drugs similarly transport statins, and we note an overlap with gene expression changes in the pathways related to native function of SLCO1B1. We included this study to expose potential overlap between pharmacokinetic and pharmacodynamics effects of gene polymorphisms and drug-induced expression changes.
   iii. Relevance of the perturbation to the physiology of the side effect pathology: Abnormal lipid oxidation state has been shown to be a predisposing factor for drug-induced myotoxicity[96].
   iv. Perturbation associated with the pathology: Higher levels of prostaglandin E2 were found in horses with halothane-induced myotoxicity compared with those not experiencing myotoxicity[97], suggesting this perturbation is tied to the side effect occurrence.
   v. Known supplements targeted at perturbed pathways: Supplementation with essential fatty acids has been shown to alleviate asthenia in mice[98], further suggesting that there may be an overlap between drug metabolism and native metabolic function as perturbed by statins.
   vi. In vivo *occurrence of metabolic perturbation: In vivo* eicosanoid metabolism has been shown to be perturbed in muscle following simvastatin and atorvastatin treatment[99], suggesting that the metabolic gene expression perturbation is physiologically relevant in the primary tissue of interest in myotoxicity.

b. Other related genes: CYP2C8, a cytochrome P450 enzyme responsible for statin degradation, has been shown to be a susceptibility gene for rhabdomyolysis[100]. Its native function is thought to be responsible for arachidonic acid metabolism[101], and thus its function also overlaps with the observed perturbations in eicosanoid metabolism.

Warfarin-induced bleeding

a. *Drugs with side effect (corresponding frequencies):* Fluoxetine (0.22628), mitoxantrone (0.036793), pergolide (0.0055), memantine (0.0055), gabapentin (0.0032966), tranexamic acid (0.0005), risperidone (0.0005), riluzole (0.0005), paroxetine (0.0005), ofloxacin (0.0005), ketorolac (0.0005), iodixanol (0.0005), fluvoxamine (0.0005), clomipramine (0.0005), ciprofloxacin (0.0005), cefuroxime (0.0005), ceftazidime (0.0005), cefotetan (0.0005), cefixime (0.0005), cefepime (0.0005), amiodarone (0.0005), valproic acid (0.0004375), propofol (0.00033333)
b. *Observed Perturbations (direction) (overlaps with gene in* ***bold****):* 1D-myo-inositol 4-phosphate (up), geranyl diphosphate (down), **prostaglandin D2 (down)**, retinoyl glucuronide (down), sphingosine 1-phosphate (down)
c. Gene: Certain alleles of CYP2C9 are associated with increased risk of bleeding under treatment with warfarin[102]
    i. *Relevant perturbation:* prostaglandin D2 (down)
    ii. *Overlaps with gene pathway:* In addition to its xenobiotic-metabolizing role, CYP2C9 is involved in the metabolism of native substrates including eicosanoids[103], and thus overlaps with the observed down-regulation centered around prostaglandin D2.
    iii. *Relevance of the perturbation to the physiology of the side effect pathology:* One of the well-characterized functions of prostaglandin is in regulating the platelet clotting response[104], and thus perturbations to this pathway could have clear significance to the risk of hemorrhage under warfarin treatment.
    iv. *Perturbation associated with the pathology:* Deficiency of essential fatty acids has been shown to result in clotting deficiency in neonates[105].
    v. *Known supplements targeted at perturbed pathways:* Intake of n-6 and n-3 fatty acids has been shown to have either a proaggregatory or an antiaggregatory effect, respectively[106].
    vi. In vivo *occurrence of metabolic perturbation:* We did not find existing evidence of warfarin decreasing *in vivo* eicosanoid levels.

Supplementary Note 4

Description of excluded categories of side effect-susceptibility gene variants

Side effect not included in study

> *Rationale:* An insufficient number of gene expression samples (< 30) from the Connectivity Map database were from drugs with a particular side effect, and so we were unable to generate predictions for the side effect.

Metabolic pathway not included in study

> *Rationale:* Certain metabolic pathways were 'off' during the MetChange algorithm analysis, due to the assumptions of cell function and media constraints, and hence no predictions were made in these pathways. Notably, this includes heme synthesis and degradation.

Genes without known or overlapping metabolic function

> *Rationale:* In cases where the gene has unknown or non-specific links to metabolism, we were unable to associate the gene with a particular pathway, and no comparison could be made. This includes pharmacokinetic genes that had no known overlapping native function, such as thiopurine methyltransferase.

Side effects with pathology manifested in enucleated cells

> *Rationale:* Genes with polymorphisms were linked to red blood cell anemias in certain drugs. However, as the red blood cell is enucleated and thus would experience no direct drug-induced gene expression changes, we excluded these cases from comparison with side effect -linked gene expression changes. We do not exclude the possibility that nucleated progenitor cells may experience drug-induced expression changes relevant to the pathology (see ribavirin-induced anemia), but it is more difficult to make meaningful hypotheses about such effects, and therefore we ignore them in these cases.

Gene polymorphism likely to impact side effect occurrence through altered drug pharmacokinetics only rather than altered metabolic gene expression

> *Rationale:* Studies involving CYPs, drug metabolizing enzymes[107], and other drug metabolism and transport proteins were generally excluded in cases of non-specific toxicity. First, as the genes are pharmacokinetic and polymorphism affects susceptibility to multiple side effects, we deem it unlikely that the gene is related to the pathology of all such side effects, but rather the effect is manifested primarily through altered pharmacokinetics. Second, while the pharmacokinetic genes are associated with side effect susceptibility of particular drugs, our predictions for a given side effect include gene expression samples from multiple drugs, many of which are likely not metabolized by the same gene, and thus we are not likely to see any conserved gene expression change related to the particular pharmacokinetic gene associated with the drug in the genetic study.

Excluded Studies: Side effect not included in study

1) Drug-induced liver injury
    a. Gene: Various[108]
        i. Reason for exclusion: There were not enough gene expression samples (< 30) from drugs that induce liver injury to obtain a consensus signature, so no predictions were made.
2) Pamidronate and zoledronate-induced osteonecrosis of the jaw
    a. Gene: CYP2C8 is associated with susceptibility to drug-induced osteonecrosis of the jaw[109]
        i. Reason for exclusion: There were not enough gene expression samples (< 30) from drugs that induce osteonecrosis of the jaw to obtain a consensus signature, so no predictions were made. Additionally, the CYP2C8 gene is tied to metabolism of the drugs, and thus since other drugs not metabolized by CYP2C8 also cause osteonecrosis of the jaw are included, the side effect would not be expected to have a consensus signature linked to this gene.

Excluded Studies: Metabolic pathway not included in study

1) Irinotecan-induced neutropenia
    a. Gene: UGT1A1 has been shown to be associated with irinotecan-induced neutropenia[110]
        i. Reason for exclusion: Native function of UGT1A1 is thought to be primarily in bilirubin glucurosylation. We did not include the bilirubin pathway in our analysis and thus had no potential predictions for comparison. Furthermore, UGT1A1 is thought to mediate susceptibility through its role in irinotecan metabolism, so gene expression changes related to UGT1A1-related pathways are not likely to be critical to side effect incidence.
2) Methemoglobin reductase linked to methemoglobinemia and porphobilinogen deaminase deficiency linked to acute porphyric crisis[111] were similarly excluded because the hemoglobin synthesis and degradation pathway was not included in our analysis
3) Statin-induced myotoxicity
    a. Gene: ATP-binding cassette G2[112]
        i. Reason for exclusion: Altered drug transport is thought to be the primary mechanism of increased susceptibility with gene polymorphisms, and thus no overlap with induced gene expression changes is expected. Native function appears to be related to porphyrin transport[113], which is a pathway that we had excluded from analysis and thus could not examine possible overlap.

Excluded Studies: Genes without known or overlapping metabolic function

1) Succinylcholine-induced apnea
    a. Gene: Serum pseudocholinesterase has been lined to apnea induced by succinylcholine[114]
        i. Reason for exclusion: The native substrate for pseudocholinesterase is not in our model, and thus there was no overlapping pathway with which to associate the gene. Additionally, the mechanism of sensitivity is thought to be through the role of the cholinesterase in metabolizing succinylcholine, and thus we did not expect to see a causal role of gene expression changes related to choline pathways in the side effect pathogenesis.

2) 6-mercaptopurine-induced hematological toxicity and cisplatin-induced ototoxicity
    a. Gene: TPMT, encoding thiopurine methyltransferase, is associated with toxicity of the chemotherapeutic agent 6-mercaptopurine[115]. Also, variants of TPMT have been associated with cisplatin-induced hearing loss[82].
        i. Reason for exclusion: The effect of TPMT polymorphisms on 6-mercaptopurine toxicity is likely primarily related to 6-mercaptopurine metabolism, but the enzyme has no native substrate identified to our knowledge[116], and thus we do not have an overlapping pathway.
3) Citalopram-induced blurry vision
    a. Gene: Emid2 is associated with citalopram-induced visual and hearing defects, including blurry vision[117]
        i. Reason for exclusion: Function of Emid2 is not fully elucidated and no clear ties to metabolism could be found for comparison[118]
4) Nevirapine-induced rash
    b. Gene: CCHCR1 has been shown to be associated with nevirapine-induced rash[119]
        i. Reason for exclusion: No clear tie between CCHCR1 and a particular metabolic pathway could be found.
5) Drug-induced cardiac arrhythmia
    c. Gene: CERKL (ceramide kinase-like protein) is associated with iloperidone-induced QT prolongation[120]
        i. Reason for exclusion: CERKL has an unclear function and thus we were unable to determine whether the gene has ties to metabolic function
    d. Gene: HERG, a potassium channel, has been shown to be associated with drug-induced torsades de pointes[121]
        i. Reason for exclusion: As a potassium channel, links between HERG and metabolism are non-specific and thus the case was excluded.
    e. Gene: KvLQT1, a potassium channel, has been shown to be associated with long QT syndrome[122]
        i. Reason for exclusion: As a potassium channel, links between KvLQT1 and metabolism are non-specific and thus the case was excluded.
    f. Gene: SCN5A, a calcium channel, has been shown to be associated with long QT syndrome[123]
        i. Reason for exclusion: As a calcium channel, links between SCN5A and metabolism are non-specific and thus the case was excluded.
6) Anesthetic and muscle relaxant-induced malignant hyperthermia
    g. Gene: RYR1, a ryanodine receptor functioning as a calcium channel, has been associated with drug-induced malignant hypothermia[124]
        i. Reason for exclusion: Links between RYR1 and metabolism are unclear and could not be tied to a particular pathway
7) Contraceptive-induced venous thrombosis
    h. Gene: Factor V, a coagulation factor, has been associated with venous thrombosis in patients taking oral contraceptives[125]
        i. Reason for exclusion: Coagulation is outside of the scope of the metabolism and so no clear overlap could be established.

8) Doxorubicin-induced cardiotoxicity
    i. Gene: Top2b, a topoisomerase, has been shown be protective from doxorubicin-induced heart failure[126]
        i. Reason for exclusion: Links between Top2b and metabolism are not direct, and thus we could not associate the gene with a particular metabolic pathway.
9) Antipsychotic-induced weight gain
    j. Gene: Various genes, including MEIS2, PRKAR2B, GPR98, FHOD3, RNF144A, ASTN2, SOX5, and ATF7IP2, were found to be associated with metabolic side effects of antipsychotics in a genome-wide association study[127]
        i. Reason for exclusion: While MEIS2 and PRKAR2B are reported as having ties to metabolism, no specific metabolic pathway could be identified as related to these genes.
10) Epirubicin-induced leukopenia/neutropenia
    k. Gene: MCPH1, a gene thought to be associated with DNA damage response[128], was associated with epirubicin-induced leukopenia[129]
        i. Reason for exclusion: Beyond a generic role as a DNA damage response and cell cycle checkpoint protein, links between MCPH1 and metabolism are unknown.
11) Abacavir-induced hypersensitivity
    a. Gene: HLA-B*5701 was identified as a risk factor for abacavir-induced hypersensitivity[130]
        i. Reason for exclusion: Links between metabolism and immune hypersensitivity are non-specific and could not be tied to a particular pathway
12) For reviews on other studies correlated drug toxicity with immune gene polymorphisms: see reviews[131-133].

Excluded Studies: Pathology manifested in enucleated cell

1) Drug-induced anemia
    a. Gene: Variants of ITPA have been shown to protect against ribavirin-induced anemia[134] via alterations of ITP levels in the red blood cell[135]
        i. Reason for exclusion: The pathology of hemolytic anemia is manifested at the level of the red blood cell, which is enucleated and thus is not expected to have a gene expression response relevant to drug action. That said, we did notice an increase of ITPA expression that was greatest in the HL-60 hematopoietic line, which, if relevant to the side effect pathology, would be consistent with the protective effect of ITPA deficiency. Thus, we note the possibility that ITPA expression could be increased in RBC progenitor cells as a response to ribavirin treatment, dysregulating nucleotide levels and increasing susceptibility to hemolytic anemia. This is merely conjecture, however, given that the level of ITPA activity has not been compared in ribavirin-treated and control RBC samples, to our knowledge.
    b. Gene: GAPD deficiency is classically associated with drug-induced anemia[136]
        i. Reason for exclusion: Pathology manifested primarily through oxidative stress generated from a pentose phosphate pathway deficiency[136]

Excluded Studies: Gene polymorphism likely to impact side effect occurrence through altered drug pharmacokinetics only rather than altered metabolic gene expression

1) Methotrexate-induced toxicities
   a. Gene: MTHFR, methylenetetrahydrofolate reductase, is associated with various methotrexate-induced toxicities[137]
      i. Reason for exclusion: Toxicity includes multiple pathologies, and thus could not be tied to a particular side effect. It is assumed therefore that the effect of polymorphisms of MTHFR on side effect incident is primarily related to pharmacokinetic effect, and therefore side effect-linked gene expression changes would not be expected to overlap with the native pathway of MTHFR
2) Fluorouracil-induced toxicity
   a. Gene: Dihydropyrimidine dehydrogenase, DPYD, deficiency has been shown to be associated with 5-fluorouracil toxicity[138]
      i. Reason for exclusion: Toxicity associated with fluorouracil is non-specific and occurs through a variety of pathologies[139], and thus we could not tie it to a particular side effect. For this reason, it is assumed that DYPD affects primarily the pharmacokinetics of the drug, and thus we do not expect a functional overlap between DYPD and gene expression changes associated with a particular side effect.
3) N-acetyltransferase-linked toxicities
   a. Gene: N-acetyltransferases are linked to various toxicities in various drugs[140]
      i. Reason for exclusion: Due to the fact that multiple side effects are associated with polymorphisms in the same gene, we assume that the effect of polymorphisms on susceptibility is primarily through their effect on metabolism of the drugs.

Supplementary References

1. Malhotra, A.K. et al. Association between common variants near the melanocortin 4 receptor gene and severe antipsychotic drug-induced weight gain. *Arch Gen Psychiatry* 69, 904-912 (2012).
2. Piccinetti, C.C. et al. Appetite regulation: the central role of melatonin in Danio rerio. *Horm Behav* 58, 780-785 (2010).
3. Nogueiras, R. et al. The central melanocortin system directly controls peripheral lipid metabolism. *J Clin Invest* 117, 3475-3488 (2007).
4. Williams, K.W., Scott, M.M. & Elmquist, J.K. From observation to experimentation: leptin action in the mediobasal hypothalamus. *Am J Clin Nutr* 89, 985S-990S (2009).
5. Reiter, R.J., Tan, D.X., Korkmaz, A. & Ma, S. Obesity and metabolic syndrome: association with chronodisruption, sleep deprivation, and melatonin suppression. *Ann Med* 44, 564-577 (2012).
6. Raskind, M.A., Burke, B.L., Crites, N.J., Tapp, A.M. & Rasmussen, D.D. Olanzapine-induced weight gain and increased visceral adiposity is blocked by melatonin replacement therapy in rats. *Neuropsychopharmacology* 32, 284-288 (2007).
7. Cremer-Bartels, G., Ebels, I., Sykes, J.E. & de Moree, A. Effects of retinal and pineal low molecular weight fractions and antipsychotic drugs on hydroxyindole-O-methyltransferase. *J Neural Transm* 58, 107-119 (1983).
8. Wagner, S. et al. A broad role for the zinc finger protein ZNF202 in human lipid metabolism. *J Biol Chem* 275, 15685-15690 (2000).
9. Porsch-Ozcurumez, M. et al. The zinc finger protein 202 (ZNF202) is a transcriptional repressor of ATP binding cassette transporter A1 (ABCA1) and ABCG1 gene expression and a modulator of cellular lipid efflux. *J Biol Chem* 276, 12427-12433 (2001).
10. Aberg, K. et al. Genomewide association study of movement-related adverse antipsychotic effects. *Biol Psychiatry* 67, 279-282 (2010).
11. Drago, A., Crisafulli, C. & Serretti, A. The genetics of antipsychotic induced tremors: a genome-wide pathway analysis on the STEP-BD SCP sample. *Am J Med Genet B Neuropsychiatr Genet* 156B, 975-986 (2011).
12. Dickson, D.W. et al. Neuropathological assessment of Parkinson's disease: refining the diagnostic criteria. *Lancet Neurol* 8, 1150-1157 (2009).
13. Rappley, I. et al. Lipidomic profiling in mouse brain reveals differences between ages and genders, with smaller changes associated with alpha-synuclein genotype. *J Neurochem* 111, 15-25 (2009).
14. Ruiperez, V., Darios, F. & Davletov, B. Alpha-synuclein, lipids and Parkinson's disease. *Prog Lipid Res* 49, 420-428 (2010).
15. Jenner, P. Oxidative stress in Parkinson's disease. *Ann Neurol* 53 Suppl 3, S26-36; discussion S36-28 (2003).
16. Cheng, D. et al. Lipid pathway alterations in Parkinson's disease primary visual cortex. *PLoS ONE* 6, e17299 (2011).
17. de Lau, L.M. et al. Dietary fatty acids and the risk of Parkinson disease: the Rotterdam study. *Neurology* 64, 2040-2045 (2005).
18. Shchepinov, M.S. et al. Isotopic reinforcement of essential polyunsaturated fatty acids diminishes nigrostriatal degeneration in a mouse model of Parkinson's disease. *Toxicol Lett* 207, 97-103 (2011).
19. Bousquet, M. et al. Beneficial effects of dietary omega-3 polyunsaturated fatty acid on toxin-induced neuronal degeneration in an animal model of Parkinson's disease. *FASEB J* 22, 1213-1225 (2008).
20. Evans, D.R. et al. Red blood cell membrane essential fatty acid metabolism in early psychotic patients following antipsychotic drug treatment. *Prostaglandins Leukot Essent Fatty Acids* 69, 393-399 (2003).
21. Tan, E.C., Chong, S.A., Mahendran, R., Dong, F. & Tan, C.H. Susceptibility to neuroleptic-induced tardive dyskinesia and the T102C polymorphism in the serotonin type 2A receptor. *Biol Psychiatry* 50, 144-147 (2001).
22. Sandyk, R. & Fisher, H. The relationship of serotonin metabolism and melatonin secretion to the pathophysiology of tardive dyskinesia. *Int J Neurosci* 48, 133-136 (1989).
23. Creed, M.C., Hamani, C., Bridgman, A., Fletcher, P.J. & Nobrega, J.N. Contribution of decreased serotonin release to the antidyskinetic effects of deep brain stimulation in a rodent model of tardive dyskinesia: comparison of the subthalamic and entopeduncular nuclei. *J Neurosci* 32, 9574-9581 (2012).

24. Rosengarten, H., Bartoszyk, G.D., Quartermain, D. & Lin, Y. The effect of chronic administration of sarizotan, 5-HT1A agonist/D3/D4 ligand, on haloperidol-induced repetitive jaw movements in rat model of tardive dyskinesia. *Prog Neuropsychopharmacol Biol Psychiatry* 30, 273-279 (2006).
25. Sirota, P., Mosheva, T., Shabtay, H., Giladi, N. & Korczyn, A.D. Use of the selective serotonin 3 receptor antagonist ondansetron in the treatment of neuroleptic-induced tardive dyskinesia. *Am J Psychiatry* 157, 287-289 (2000).
26. Nasrallah, H.A., Smith, R.E., Dunner, F.J. & McCalley-Whitters, M. Serotonin precursor effects in tardive dyskinesia. *Psychopharmacology (Berl)* 77, 234-235 (1982).
27. Khan, A., Haleem, M.A. & Haleem, D.J. Dopamine and Serotonin Metabolism in the Dorsal and Ventral Striatum of Haloperidol-induced Tardive Dyskinesia Model in Rats. *Journal of the Chemical Society of Pakistan* 30, 410-416 (2008).
28. Steen, V.M., Lovlie, R., MacEwan, T. & McCreadie, R.G. Dopamine D3-receptor gene variant and susceptibility to tardive dyskinesia in schizophrenic patients. *Mol Psychiatry* 2, 139-145 (1997).
29. Segman, R.H. et al. Association of dopaminergic and serotonergic genes with tardive dyskinesia in patients with chronic schizophrenia. *Pharmacogenomics J* 3, 277-283 (2003).
30. de Angelis, L. Ascorbic acid and atypical antipsychotic drugs: modulation of amineptine-induced behavior in mice. *Brain Res* 670, 303-307 (1995).
31. Deshpande, C., Dhir, A. & Kulkarni, S.K. Antagonistic activity of ascorbic acid (vitamin C) on dopaminergic modulation: apomorphine-induced stereotypic behavior in mice. *Pharmacology* 77, 38-45 (2006).
32. Michael, N., Sourgens, H., Arolt, V. & Erfurth, A. Severe tardive dyskinesia in affective disorders: treatment with vitamin E and C. *Neuropsychobiology* 46 Suppl 1, 28-30 (2002).
33. Dakhale, G.N., Khanzode, S.D., Khanzode, S.S. & Saoji, A. Supplementation of vitamin C with atypical antipsychotics reduces oxidative stress and improves the outcome of schizophrenia. *Psychopharmacology (Berl)* 182, 494-498 (2005).
34. Suboticanec, K., Folnegovic-Smalc, V., Korbar, M., Mestrovic, B. & Buzina, R. Vitamin C status in chronic schizophrenia. *Biol Psychiatry* 28, 959-966 (1990).
35. Dakhale, G., Khanzode, S., Saoji, A., Khobragade, L. & Turankar, A. Oxidative damage and schizophrenia: the potential benefit by atypical antipsychotics. *Neuropsychobiology* 49, 205-209 (2004).
36. Ramirez, A.H. et al. Novel rare variants in congenital cardiac arrhythmia genes are frequent in drug-induced torsades de pointes. *Pharmacogenomics J* (2012).
37. London, B. et al. Mutation in glycerol-3-phosphate dehydrogenase 1 like gene (GPD1-L) decreases cardiac Na+ current and causes inherited arrhythmias. *Circulation* 116, 2260-2268 (2007).
38. Benjamin, E.J. et al. Variants in ZFHX3 are associated with atrial fibrillation in individuals of European ancestry. *Nat Genet* 41, 879-881 (2009).
39. Valdivia, C.R., Ueda, K., Ackerman, M.J. & Makielski, J.C. GPD1L links redox state to cardiac excitability by PKC-dependent phosphorylation of the sodium channel SCN5A. *Am J Physiol Heart Circ Physiol* 297, H1446-1452 (2009).
40. Kim, T.S. et al. The ZFHX3 (ATBF1) transcription factor induces PDGFRB, which activates ATM in the cytoplasm to protect cerebellar neurons from oxidative stress. *Dis Model Mech* 3, 752-762 (2010).
41. Rozanski, G.J. & Xu, Z. Glutathione and K(+) channel remodeling in postinfarction rat heart. *Am J Physiol Heart Circ Physiol* 282, H2346-2355 (2002).
42. Brown, D.A. & O'Rourke, B. Cardiac mitochondria and arrhythmias. *Cardiovasc Res* 88, 241-249 (2010).
43. Aon, M.A., Cortassa, S., Marban, E. & O'Rourke, B. Synchronized whole cell oscillations in mitochondrial metabolism triggered by a local release of reactive oxygen species in cardiac myocytes. *J Biol Chem* 278, 44735-44744 (2003).
44. Aggarwal, N.T. & Makielski, J.C. Redox Control of Cardiac Excitability. *Antioxid Redox Signal* (2012).
45. Duan, J. & Moffat, M.P. Potential cellular mechanisms of hydrogen peroxide-induced cardiac arrhythmias. *J Cardiovasc Pharmacol* 19, 593-601 (1992).
46. Sethi, R. et al. Antiarrhythmic effects of some antioxidant vitamins in rats injected with epinephrine. *Cardiovasc Toxicol* 9, 177-184 (2009).

47. Sochman, J., Kolc, J., Vrana, M. & Fabian, J. Cardioprotective effects of N-acetylcysteine: the reduction in the extent of infarction and occurrence of reperfusion arrhythmias in the dog. *Int J Cardiol* 28, 191-196 (1990).
48. Chahine, R. & Feng, J. Protective effects of taurine against reperfusion-induced arrhythmias in isolated ischemic rat heart. *Arzneimittelforschung* 48, 360-364 (1998).
49. Hanna, J. et al. Protective effect of taurine against free radicals damage in the rat myocardium. *Exp Toxicol Pathol* 56, 189-194 (2004).
50. Tan, D.X. et al. Ischemia/reperfusion-induced arrhythmias in the isolated rat heart: prevention by melatonin. *J Pineal Res* 25, 184-191 (1998).
51. Morisco, C., Trimarco, B. & Condorelli, M. Effect of coenzyme Q10 therapy in patients with congestive heart failure: a long-term multicenter randomized study. *Clin Investig* 71, S134-136 (1993).
52. El-Demerdash, E. & Mohamadin, A.M. Does oxidative stress contribute in tricyclic antidepressants-induced cardiotoxicity? *Toxicol Lett* 152, 159-166 (2004).
53. Jamshidi, Y. et al. Common variation in the NOS1AP gene is associated with drug-induced QT prolongation and ventricular arrhythmia. *J Am Coll Cardiol* 60, 841-850 (2012).
54. Fang, M. et al. Dexras1: a G protein specifically coupled to neuronal nitric oxide synthase via CAPON. *Neuron* 28, 183-193 (2000).
55. Danson, E.J., Choate, J.K. & Paterson, D.J. Cardiac nitric oxide: emerging role for nNOS in regulating physiological function. *Pharmacol Ther* 106, 57-74 (2005).
56. Tamargo, J., Caballero, R., Gomez, R. & Delpon, E. Cardiac electrophysiological effects of nitric oxide. *Cardiovasc Res* 87, 593-600 (2010).
57. van Obbergh, L., Vallieres, Y. & Blaise, G. Cardiac modifications occurring in the ascitic rat with biliary cirrhosis are nitric oxide related. *J Hepatol* 24, 747-752 (1996).
58. Tavakoli, S. et al. Reduced susceptibility to epinephrine-induced arrhythmias in cirrhotic rats: the roles of nitric oxide and endogenous opioid peptides. *J Hepatol* 46, 432-439 (2007).
59. Altug, S. et al. The role of nitric oxide in digoxin-induced arrhythmias in guinea-pigs. *Pharmacol Toxicol* 84, 3-8 (1999).
60. Sansone, G.R., Matin, A., Wang, S.F., Bouboulis, D. & Frieri, M. Theophylline inhibits the production of nitric oxide by peripheral blood mononuclear cells from patients with asthma. *Ann Allergy Asthma Immunol* 81, 90-95 (1998).
61. Wegener, G., Volke, V., Harvey, B.H. & Rosenberg, R. Local, but not systemic, administration of serotonergic antidepressants decreases hippocampal nitric oxide synthase activity. *Brain Res* 959, 128-134 (2003).
62. Park, K.H., Kwok, S.M., Sharon, C., Baerga, R. & Sesti, F. N-Glycosylation-dependent block is a novel mechanism for drug-induced cardiac arrhythmia. *FASEB J* 17, 2308-2309 (2003).
63. Zijlstra, W.G., Brunsting, J.R., ten Hoor, F. & Vergroesen, A.J. Prostaglandin E 1 and cardiac arrhythmia. *Eur J Pharmacol* 18, 392-395 (1972).
64. Kelliher, G.J. & Glenn, T.M. Effect of prostaglandin E1 on ouabain-induced arrhythmia. *Eur J Pharmacol* 24, 410-414 (1973).
65. Stavrou, B.M., Sheridan, D.J. & Flores, N.A. Contribution of nitric oxide and prostanoids to the cardiac electrophysiological and coronary vasomotor effects of diadenosine polyphosphates. *J Pharmacol Exp Ther* 298, 531-538 (2001).
66. Friedrich, T., Lichey, J., Nigam, S., Priesnitz, M. & Wegscheider, K. Follow-up of prostaglandin plasma levels after acute myocardial infarction. *Am Heart J* 109, 218-222 (1985).
67. Oh, J., Ban, M.R., Miskie, B.A., Pollex, R.L. & Hegele, R.A. Genetic determinants of statin intolerance. *Lipids Health Dis* 6, 7 (2007).
68. Marcoff, L. & Thompson, P.D. The role of coenzyme Q10 in statin-associated myopathy: a systematic review. *J Am Coll Cardiol* 49, 2231-2237 (2007).
69. Deichmann, R., Lavie, C. & Andrews, S. Coenzyme q10 and statin-induced mitochondrial dysfunction. *Ochsner J* 10, 16-21 (2010).
70. Gempel, K. et al. The myopathic form of coenzyme Q10 deficiency is caused by mutations in the electron-transferring-flavoprotein dehydrogenase (ETFDH) gene. *Brain* 130, 2037-2044 (2007).

71. Lalani, S.R. et al. Isolated mitochondrial myopathy associated with muscle coenzyme Q10 deficiency. *Arch Neurol* 62, 317-320 (2005).
72. Horvath, R. et al. Coenzyme Q10 deficiency and isolated myopathy. *Neurology* 66, 253-255 (2006).
73. Langsjoen, P.H., Langsjoen, J.O., Langsjoen, A.M. & Lucas, L.A. Treatment of statin adverse effects with supplemental Coenzyme Q10 and statin drug discontinuation. *Biofactors* 25, 147-152 (2005).
74. Wyman, M., Leonard, M. & Morledge, T. Coenzyme Q10: a therapy for hypertension and statin-induced myalgia? *Cleve Clin J Med* 77, 435-442 (2010).
75. Schaars, C.F. & Stalenhoef, A.F. Effects of ubiquinone (coenzyme Q10) on myopathy in statin users. *Curr Opin Lipidol* 19, 553-557 (2008).
76. Diaz-Castro, J. et al. Coenzyme Q(10) supplementation ameliorates inflammatory signaling and oxidative stress associated with strenuous exercise. *Eur J Nutr* 51, 791-799 (2012).
77. Paiva, H. et al. High-dose statins and skeletal muscle metabolism in humans: a randomized, controlled trial. *Clin Pharmacol Ther* 78, 60-68 (2005).
78. Strey, C.H. et al. Endothelium-ameliorating effects of statin therapy and coenzyme Q10 reductions in chronic heart failure. *Atherosclerosis* 179, 201-206 (2005).
79. Avis, H.J. et al. Rosuvastatin lowers coenzyme Q10 levels, but not mitochondrial adenosine triphosphate synthesis, in children with familial hypercholesterolemia. *J Pediatr* 158, 458-462 (2011).
80. Lamperti, C. et al. Muscle coenzyme Q10 level in statin-related myopathy. *Arch Neurol* 62, 1709-1712 (2005).
81. Ghirlanda, G. et al. Evidence of plasma CoQ10-lowering effect by HMG-CoA reductase inhibitors: a double-blind, placebo-controlled study. *J Clin Pharmacol* 33, 226-229 (1993).
82. Ross, C.J. et al. Genetic variants in TPMT and COMT are associated with hearing loss in children receiving cisplatin chemotherapy. *Nat Genet* 41, 1345-1349 (2009).
83. Darrow, K.N., Simons, E.J., Dodds, L. & Liberman, M.C. Dopaminergic innervation of the mouse inner ear: evidence for a separate cytochemical group of cochlear efferent fibers. *The Journal of comparative neurology* 498, 403-414 (2006).
84. Lounkine, E. et al. Large-scale prediction and testing of drug activity on side-effect targets. *Nature* 486, 361-367 (2012).
85. Zhao, X.M. et al. Prediction of drug combinations by integrating molecular and pharmacological data. *PLoS Comput Biol* 7, e1002323 (2011).
86. Babcock, J.J., Du, F., Xu, K., Wheelan, S.J. & Li, M. Integrated analysis of drug-induced gene expression profiles predicts novel hERG inhibitors. *PLoS ONE* 8, e69513 (2013).
87. Samaras, D. et al. Effects of widely used drugs on micronutrients: a story rarely told. *Nutrition* 29, 605-610 (2013).
88. Lee, S., Lee, K.H., Song, M. & Lee, D. Building the process-drug-side effect network to discover the relationship between biological processes and side effects. *Bmc Bioinformatics* 12 Suppl 2, S2 (2011).
89. Bordbar, A. & Palsson, B.O. Using the reconstructed genome-scale human metabolic network to study physiology and pathology. *J Intern Med* 271, 131-141 (2012).
90. Peters, U. et al. Glutathione S-transferase genetic polymorphisms and individual sensitivity to the ototoxic effect of cisplatin. *Anti-cancer drugs* 11, 639-643 (2000).
91. Bordbar, A., Monk, J.M., King, Z.A. & Palsson, B.O. Constraint-based models predict metabolic and associated cellular functions. *Nat Rev Genet* 15, 107-120 (2014).
92. Law, V. et al. DrugBank 4.0: shedding new light on drug metabolism. *Nucleic Acids Res* 42, D1091-1097 (2014).
93. Toyoshiba, H., Sawada, H., Naeshiro, I. & Horinouchi, A. Similar compounds searching system by using the gene expression microarray database. *Toxicol Lett* 186, 52-57 (2009).
94. Ong, S.E. et al. Identifying the proteins to which small-molecule probes and drugs bind in cells. *Proc Natl Acad Sci U S A* 106, 4617-4622 (2009).
95. Link, E. et al. SLCO1B1 variants and statin-induced myopathy--a genomewide study. *N Engl J Med* 359, 789-799 (2008).
96. Phillips, P.S., Phillips, C.T., Sullivan, M.J., Naviaux, R.K. & Haas, R.H. Statin myotoxicity is associated with changes in the cardiopulmonary function. *Atherosclerosis* 177, 183-188 (2004).

97. Serteyn, D. et al. Equine postanaesthetic myositis: thromboxanes, prostacyclin and prostaglandin E2 production. *Vet Res Commun* 12, 219-226 (1988).
98. Hardman, W.E., Moyer, M.P. & Cameron, I.L. Consumption of an omega-3 fatty acids product, INCELL AAFA, reduced side-effects of CPT-11 (irinotecan) in mice. *Br J Cancer* 86, 983-988 (2002).
99. Laaksonen, R. et al. A systems biology strategy reveals biological pathways and plasma biomarker candidates for potentially toxic statin-induced changes in muscle. *PLoS ONE* 1, e97 (2006).
100. Marciante, K.D. et al. Cerivastatin, genetic variants, and the risk of rhabdomyolysis. *Pharmacogenet Genomics* 21, 280-288 (2011).
101. Totah, R.A. & Rettie, A.E. Cytochrome P450 2C8: substrates, inhibitors, pharmacogenetics, and clinical relevance. *Clin Pharmacol Ther* 77, 341-352 (2005).
102. Sanderson, S., Emery, J. & Higgins, J. CYP2C9 gene variants, drug dose, and bleeding risk in warfarin-treated patients: a HuGEnet systematic review and meta-analysis. *Genet Med* 7, 97-104 (2005).
103. Rettie, A.E. & Jones, J.P. Clinical and toxicological relevance of CYP2C9: drug-drug interactions and pharmacogenetics. *Annu Rev Pharmacol Toxicol* 45, 477-494 (2005).
104. Smith, J.B. The prostanoids in hemostasis and thrombosis: a review. *Am J Pathol* 99, 743-804 (1980).
105. Friedman, Z., Lamberth, E.L., Jr., Stahlman, M.T. & Oates, J.A. Platelet dysfunction in the neonate with essential fatty acid deficiency. *J Pediatr* 90, 439-443 (1977).
106. Simopoulos, A.P. Essential fatty acids in health and chronic disease. *Am J Clin Nutr* 70, 560S-569S (1999).
107. Johansson, I. & Ingelman-Sundberg, M. Genetic polymorphism and toxicology--with emphasis on cytochrome p450. *Toxicol Sci* 120, 1-13 (2011).
108. Stephens, C., Lucena, M.I. & Andrade, R.J. Genetic variations in drug-induced liver injury (DILI): resolving the puzzle. *Front Genet* 3, 253 (2012).
109. Sarasquete, M.E. et al. Bisphosphonate-related osteonecrosis of the jaw is associated with polymorphisms of the cytochrome P450 CYP2C8 in multiple myeloma: a genome-wide single nucleotide polymorphism analysis. *Blood* 112, 2709-2712 (2008).
110. Hoskins, J.M., Goldberg, R.M., Qu, P., Ibrahim, J.G. & McLeod, H.L. UGT1A1*28 genotype and irinotecan-induced neutropenia: dose matters. *J Natl Cancer Inst* 99, 1290-1295 (2007).
111. Pirmohamed, M. & Park, B.K. Genetic susceptibility to adverse drug reactions. *Trends Pharmacol Sci* 22, 298-305 (2001).
112. Niemi, M. Transporter pharmacogenetics and statin toxicity. *Clin Pharmacol Ther* 87, 130-133 (2010).
113. Krishnamurthy, P. & Schuetz, J.D. Role of ABCG2/BCRP in biology and medicine. *Annu Rev Pharmacol Toxicol* 46, 381-410 (2006).
114. Lockridge, O. Genetic variants of human serum cholinesterase influence metabolism of the muscle relaxant succinylcholine. *Pharmacol Ther* 47, 35-60 (1990).
115. Lennard, L., Lilleyman, J.S., Van Loon, J. & Weinshilboum, R.M. Genetic variation in response to 6-mercaptopurine for childhood acute lymphoblastic leukaemia. *Lancet* 336, 225-229 (1990).
116. Peng, Y. et al. Structural basis of substrate recognition in thiopurine s-methyltransferase. *Biochemistry* 47, 6216-6225 (2008).
117. Adkins, D.E. et al. Genome-wide pharmacogenomic study of citalopram-induced side effects in STAR*D. *Transl Psychiatry* 2, e129 (2012).
118. Pasaje, C.F. et al. Possible role of EMID2 on nasal polyps pathogenesis in Korean asthma patients. *BMC Med Genet* 13, 2 (2012).
119. Chantarangsu, S. et al. Genome-wide association study identifies variations in 6p21.3 associated with nevirapine-induced rash. *Clin Infect Dis* 53, 341-348 (2011).
120. Volpi, S. et al. Whole genome association study identifies polymorphisms associated with QT prolongation during iloperidone treatment of schizophrenia. *Mol Psychiatry* 14, 1024-1031 (2009).
121. Shah, R.R. Pharmacogenetic aspects of drug-induced torsade de pointes: potential tool for improving clinical drug development and prescribing. *Drug Saf* 27, 145-172 (2004).
122. Napolitano, C. et al. Evidence for a cardiac ion channel mutation underlying drug-induced QT prolongation and life-threatening arrhythmias. *J Cardiovasc Electrophysiol* 11, 691-696 (2000).

123. Wang, Q. et al. SCN5A mutations associated with an inherited cardiac arrhythmia, long QT syndrome. *Cell* 80, 805-811 (1995).
124. MacLennan, D.H. et al. The role of the skeletal muscle ryanodine receptor gene in malignant hyperthermia. *Symp Soc Exp Biol* 46, 189-201 (1992).
125. Vandenbroucke, J.P. et al. Increased risk of venous thrombosis in oral-contraceptive users who are carriers of factor V Leiden mutation. *Lancet* 344, 1453-1457 (1994).
126. Zhang, S. et al. Identification of the molecular basis of doxorubicin-induced cardiotoxicity. *Nat Med* 18, 1639-1642 (2012).
127. Adkins, D.E. et al. Genomewide pharmacogenomic study of metabolic side effects to antipsychotic drugs. *Mol Psychiatry* 16, 321-332 (2011).
128. Rai, R. et al. BRIT1 regulates early DNA damage response, chromosomal integrity, and cancer. *Cancer Cell* 10, 145-157 (2006).
129. Srinivasan, Y. et al. Genome-wide association study of epirubicin-induced leukopenia in Japanese patients. *Pharmacogenet Genomics* 21, 552-558 (2011).
130. Hughes, A.R., Brothers, C.H., Mosteller, M., Spreen, W.R. & Burns, D.K. Genetic association studies to detect adverse drug reactions: abacavir hypersensitivity as an example. *Pharmacogenomics* 10, 225-233 (2009).
131. Bugelski, P.J. Genetic aspects of immune-mediated adverse drug effects. *Nat Rev Drug Discov* 4, 59-69 (2005).
132. Daly, A.K. Using genome-wide association studies to identify genes important in serious adverse drug reactions. *Annu Rev Pharmacol Toxicol* 52, 21-35 (2012).
133. Wilke, R.A. et al. Identifying genetic risk factors for serious adverse drug reactions: current progress and challenges. *Nat Rev Drug Discov* 6, 904-916 (2007).
134. Thompson, A.J. et al. Variants in the ITPA gene protect against ribavirin-induced hemolytic anemia and decrease the need for ribavirin dose reduction. *Gastroenterology* 139, 1181-1189 (2010).
135. Hitomi, Y. et al. Inosine triphosphate protects against ribavirin-induced adenosine triphosphate loss by adenylosuccinate synthase function. *Gastroenterology* 140, 1314-1321 (2011).
136. Beutler, E. G6PD: population genetics and clinical manifestations. *Blood Rev* 10, 45-52 (1996).
137. Yang, L., Hu, X. & Xu, L. Impact of methylenetetrahydrofolate reductase (MTHFR) polymorphisms on methotrexate-induced toxicities in acute lymphoblastic leukemia: a meta-analysis. *Tumour Biol* 33, 1445-1454 (2012).
138. Mattison, L.K., Soong, R. & Diasio, R.B. Implications of dihydropyrimidine dehydrogenase on 5-fluorouracil pharmacogenetics and pharmacogenomics. *Pharmacogenomics* 3, 485-492 (2002).
139. Tuchman, M. et al. Familial pyrimidinemia and pyrimidinuria associated with severe fluorouracil toxicity. *N Engl J Med* 313, 245-249 (1985).
140. Meyer, U.A. Pharmacogenetics and adverse drug reactions. *Lancet* 356, 1667-1671 (2000).
